# Regulation of Treg cells by cytokine signaling and co-stimulatory molecules

**DOI:** 10.3389/fimmu.2024.1387975

**Published:** 2024-05-13

**Authors:** Yuan Zong, Kaihang Deng, Wai Po Chong

**Affiliations:** ^1^ School of Chinese Medicine, Hong Kong Baptist University, Hong Kong, China; ^2^ Institute for Research and Continuing Education, Hong Kong Baptist University, Shenzhen, China

**Keywords:** regulatory T cells, cytokines, signaling pathways, autoimmune diseases, tumors

## Abstract

CD4^+^CD25^+^Foxp3^+^ regulatory T cells (Tregs), a vital component of the immune system, are responsible for maintaining immune homeostasis and preventing excessive immune responses. This review explores the signaling pathways of the cytokines that regulate Treg cells, including transforming growth factor beta (TGF-β), interleukin (IL)-2, IL-10, and IL-35, which foster the differentiation and enhance the immunosuppressive capabilities of Tregs. It also examines how, conversely, signals mediated by IL-6 and tumor necrosis factor -alpha (TNF-α) can undermine Treg suppressive functions or even drive their reprogramming into effector T cells. The B7 family comprises indispensable co-stimulators for T cell activation. Among its members, this review focuses on the capacity of CTLA-4 and PD-1 to regulate the differentiation, function, and survival of Tregs. As Tregs play an essential role in maintaining immune homeostasis, their dysfunction contributes to the pathogenesis of autoimmune diseases. This review delves into the potential of employing Treg-based immunotherapy for the treatment of autoimmune diseases, transplant rejection, and cancer. By shedding light on these topics, this article aims to enhance our understanding of the regulation of Tregs by cytokines and their therapeutic potential for various pathological conditions.

## 1 Introduction

CD4^+^CD25^+^Foxp3^+^ regulatory T cells (Tregs) are immunoregulatory cells that express the master transcription factor forkhead box protein 3 (Foxp3) (1). Tregs exhibit persistent and high expression of the interleukin (IL)-2 receptor alpha chain or CD25, as IL-2 is crucial for their survival and proper functioning (2). Tregs refer to those T cell subsets that can regulate or suppress the overreaction of the immune system. Although Tregs account for only 3–10% of the peripheral CD4^+^ T cell population, they are crucial for maintaining immune tolerance by suppressing the activation, proliferation, and function of effector immune cells. They secrete anti-inflammatory cytokines such as IL-10, IL-35, and transforming growth factor beta (TGF-β) to inhibit immune cells in a contact-independent manner (3, 4). Additionally, Tregs possess a high level of CD25 surface expression, which leads to the consumption of IL-2 in the surrounding environment. This consumption helps restrict the proliferation and activation of effector T cells (Teffs) (5). Tregs also suppress immune cells through contact-dependent mechanisms involving co-stimulatory molecules, such as cytotoxic T lymphocyte–associated protein 4 (CTLA-4), programmed cell death protein 1 (PD-1), and programmed death ligand 1 (PD-L1) (6–10). Through these regulatory mechanisms, Tregs play a crucial role in inhibiting the activity of immune cells, ensuring the balance of the immune system, and preventing the occurrence of excessive immune responses and the development of autoimmune diseases (11, 12).

Tregs can be classified based on their developmental origins (13). Thymus-derived Tregs (tTregs) develop in the thymus, while a small proportion of Tregs is derived from conventional T cells (Tconvs) or peripherally derived Tregs (pTregs) and matures under specific conditions, such as exposure to microbial antigens in the intestinal mucosa (14). In the presence of specific cytokines (i.e., TGF-β and IL-2), antigen stimulation *in vitro* can induce the expression of Foxp3 in Tconvs, which exhibit phenotypic and functional characteristics similar to those of tTregs and pTregs and are called inducible Tregs (iTregs) (15). Subsets of Tregs in peripheral blood exhibit T helper–like characteristics, meaning they share chemokine receptor and transcription factor expression with T helper cells (16). Examples include Th1-Tregs (CXCR3^+^ T-BET^+^ Foxp3^+^ Tregs) and Th2-Tregs (CCR8^+^ GATA3^+^ Foxp3^+^ Tregs) (17). Another type of T helper–like Treg cell is the follicular regulatory T (Tfr) cell, which suppresses follicular helper T (Tfh) cells. Tfr cells play a critical role in germinal center reactions and antibody production, and defects in Tfr cells lead to antibody accumulation and the occurrence of widespread autoimmune diseases (18–20). These Treg subsets are summarized in **Table 1**.

**Table 1:** Treg subtypes and their specific markers and characteristics.

Treg Subtype	Origin	Main Markers
pTregs (21, 22)	Peripheral	GATA3, IRF4, RORC, TBX21, HELIOS
tTregs (21, 23)	Thymus	CCR7, CD45RA, CD31, SELL, NRP1
iTregs (21, 24, 25)	Peripheral	Treg-specific demethylated region (TSDR)
Th1-Tregs (26)	Peripheral	CXCR, Tbet
Th2-Tregs (27, 28)	Peripheral	IL-4, IL-13, IRF4 CCR6-, CXCR3-, CCR4, GATA3
Tr1 (29, 30)	Peripheral	IL-10, CD49b, Lag3, Foxp3-
Tr35 (31)	Peripheral	IL-35, Foxp3-

The complex interactions among T cell subsets are characterized by diverse functional dynamics. Both iTregs and pTregs typically differentiate from Tconvs, a step critical for peripheral tolerance. It is essential to distinguish tTregs from pTregs and iTregs as they display distinct roles in maintaining central tolerance. The transcription factor Helios has been identified as a biomarker for stable tTregs (32). Additionally, research has uncovered specialized subsets of Tregs, such as Th1-Tregs and Th2-Tregs, each characterized by distinct functions tailored to modulate Th1 and Th2 responses, respectively (33, 34). Indeed, the Treg compartment exhibits a degree of plasticity that enables Tregs to modulate their suppressive functions according to the surrounding microenvironment. For example, interferon gamma (IFN-γ) or IL-27 induce Th1-Tregs with expression of Th1-related molecules, namely chemokine (C-X-C motif) receptor 3 (CXCR3) and T-box gene expressed in T cells (T-bet) (35), which can migrate to sites of Th1 inflammation and suppress Th1 cells effectively (36). Th2-specific Tregs are tailored to suppress Th2 responses, which are associated with allergic inflammation and characterized by Th2 cytokines like IL-4, IL-5, and IL-13, as well as GATA binding protein 3(GATA3) (37, 38).

The development and function of Tregs are heavily reliant on cytokines and co-stimulatory molecules. Exploring their regulatory mechanisms in Tregs is essential for gaining a deeper understanding of immune regulation, the development of related diseases, and the potential for new immunotherapeutic approaches. This review focuses on the role of these signaling pathways in Tregs and their potential implications for future therapeutic strategies.

## 2 Treg-promoting cytokines

### 2.1 TGF-β

**Figure 1:**
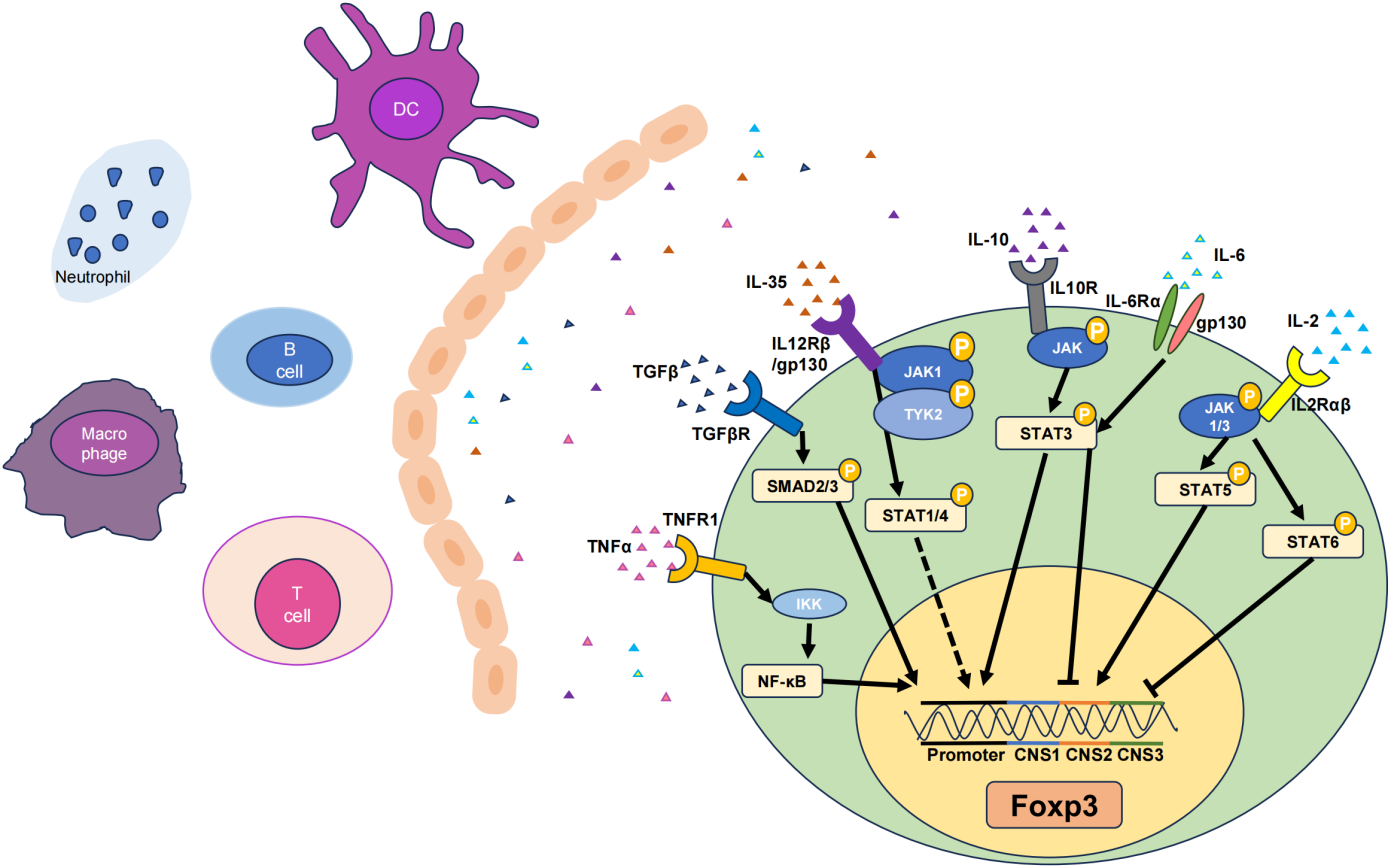
Six cytokines that promote Tregs. The TNF-α, TGF-β, IL-6, IL-35, IL-10, and IL-2 pathways can influence their respective receptors to varying degrees, thereby activating different signaling pathways and ultimately upregulating the transcription levels of Foxp3, resulting in increases in the number and stability of Tregs. Among them, there are gene loci that represent the corresponding signaling pathways and functions of different cytokines. Solid lines indicate pathways that have been functionally analysed, while dashed lines represent the effect on the Foxp3 gene without specific key loci identified. JAK, Janus-family tyrosine kinase; CNS, non-coding sequence; TYK2, tyrosine kinase 2. Arrows represent signal path direction, dashed lines represent ambiguity, and horizontal lines represent suppression.

TGF-β is a multifunctional cytokine produced by macrophages and T cells that plays a critical role in immune regulation. TGF-β exerts its immunoregulatory effects primarily by modulating the development and function of T cell subsets (39). TGF-β induces the expression of Foxp3, a key transcription factor for Tregs, by activating the suppressor of mothers against decapentaplegic (SMAD) signaling pathway (40). This promotes the generation of Tregs, which suppress inflammation and prevent autoimmune reactions. Members of the TGF-β family cooperate with receptors as ligands to form receptor complexes and activate receptor-regulated SMAD (R-SMAD), which cooperates with common mediator SMAD (Co-SMAD) to enter the nucleus. By identifying different SMAD-binding proteins and forming complexes, the specific expression of various target genes is regulated, and multiple biological effects of TGF-β ultimately occur (41). The SMAD family of proteins is the first set of signaling molecules involved in TGF-β signal transduction (40), and this signaling is tightly regulated by inhibitory SMAD (I-SMAD i.e., SMAD6 and SMAD7). The N-terminal domains of SMAD6 and SMAD7 share only 37% homology, while chimeras containing the SMAD7 N-terminal domain and SMAD6 MH2 domain inhibit TGF-β signaling pathways in the same manner as SMAD7 (42). SMAD6 is biased to inhibit signaling pathways induced by the BMP type I receptors ALK-3 and ALK-6. SMAD6^−/−^ mice can develop a variety of cardiovascular diseases (43). SMAD7 inhibits the formation of SMAD complexes and prevents the phosphorylation of SMAD2 and SMAD3, thereby interrupting the signaling cascade and influencing TGF-β signaling and Treg induction (**Figure 1**) (44).

TGF-β also plays an important regulatory role in the balance between Treg and Th17 cell differentiation (45). Endogenous TGF-β, along with inflammatory mediators such as IL-6, IL-21, and IL-23, inhibits Foxp3 expression and initiates the differentiation pathway of retinoic acid receptor–related orphan receptor gamma-t (RORγt)–mediated Th17 cells (46). As IL-6 levels decrease during the late phase of inflammation, TGF-β alone promotes the expression of Foxp3 for Treg differentiation and suppresses RORγt to limit Th17 cells, thus maintaining Treg function and controlling the effector cell response for the termination of the immune response (47, 48). Within the cell, the TGF-β/SMAD signaling pathway promotes the expression of Foxp3. SMAD3 can cooperate with nuclear factor of activated T cells (NFAT) to enhance histone acetylation in the Foxp3 enhancer region, thereby inducing Foxp3 transcription (49). Compared to controls, SMAD3^−/−^ mice exhibit a significant reduction in the quantity of Foxp3 induced by TGF-β (50).

The combination of TGF-β and the immunosuppressant rapamycin can significantly promote the proliferation of Tregs, indicating that rapamycin relies largely on TGF-β to exert its immunosuppressive effect (51). Rapamycin is a 32-ring azotriene-containing macrolide that inhibits immunity by inhibiting IL-2 signal transduction by blocking mTOR which is important in IL-2 receptor signaling (52). The IL-2 receptor signaling also leads to the activation of the PI3K/Akt pathway. Akt then activates mTOR by inhibiting the tuberous sclerosis complex (TSC1/TSC2), which normally suppresses mTOR activity. The rapamycin the FK506-binding protein 12 (FKBP12) complex binds to mechanistic target of rapamycin complex1 (mTORC1), inhibits the mammalian target protein of rapamycin (mTOR) pathway, and induces immunosuppression (53). By inhibiting mTORC1, rapamycin effectively blocks the downstream effects of mTOR activation, including protein synthesis and cell cycle progression, which are necessary for T cell proliferation. High-dose rapamycin can promote the proliferation of Tregs by inhibiting the mTOR signaling pathway, thereby significantly inhibiting the progression of experimental autoimmune encephalomyelitis (EAE) in a model and eventually mitigating the incidence and EAE clinical scores of each stage (early onset, peak, and remission) (54).

Many preclinical studies have shown that blocking TGF-β signaling is an effective anti-tumor treatment that can reduce Treg-mediated immunosuppression, increase CD8^+^ T cell cytotoxicity, promote T cell penetration into the center of the tumor, and thus contribute to strong anti-tumor immunity and tumor regression (55). TGF-β suppresses the immune system by modulating the function of immune cell classes in the tumor microenvironment (TME) (56, 57).

### 2.2 IL-2

IL-2 is a cytokine mainly produced by activated CD4^+^ T cells, particularly Th1 subsets (58). In the thymus and peripheral lymphoid organs, IL-2 signaling promotes the differentiation and development of Tregs by binding to the high-affinity IL-2 receptor (IL-2R), which is a heterotrimer consisting of the IL-2R(α/β/γ) subunits. This leads to an increase in Foxp3 gene expression in T cells, promoting the differentiation of Foxp3^+^ Tregs (59). Upon IL-2 binding to its receptor, the receptor-associated JAKs, specifically JAK1 and JAK3, are activated. JAK1 is associated with the IL-2Rβ chain, and JAK3 is closely linked with the IL-2Rγ chain (60). The activation of JAK kinases is initiated by their phosphorylation, which subsequently enables them to phosphorylate IL-2Rβ and other downstream signaling molecules. This phosphorylation creates docking sites for signaling molecules containing SH2 domains. Specifically, STAT5 is attracted to the phosphorylated IL-2Rβ chain. Within the STAT5 family, there are two distinct proteins, STAT5A and STAT5B, both of which undergo phosphorylation by the action of JAK kinases (61). Once phosphorylated, STAT5 proteins form homodimers or heterodimers, dissociate from the receptor, and translocate to the nucleus where they bind to specific DNA elements and promote the transcription of target genes (62). Previous studies have revealed that IL-2 signaling activates STAT5 and promotes Foxp3 expression by binding to the intronic enhancer element within conserved non-coding sequence 2 (CNS2) of the Foxp3 gene cluster (**Figure 1**) (63). This process is essential for maintaining Foxp3 expression in mature Treg cells. Phosphatase 2A (PP2A) is a negative regulator of IL-2 production in Teffs (64) and prevents IL-2Rβ from being clipped from the cell surface by restricting the activity of ADAM metallopeptidase domain 10 (ADAM10) in Tregs, thereby achieving effective IL-2R signaling and ultimately affecting Tregs (65).

IL-2 and IL-2/anti–IL-2 mAb immunocomplexes have been shown to have therapeutic efficacy against autoimmune diseases in preclinical studies via Treg promotions. The complex of IL-2/JES6 (IL-2 and JES6–1 mAb) can selectively expand Tregs for the suppression of autoimmune diseases in EAE (66). With increasing research on the immune regulatory mechanism of low-dose IL-2, IL-2 has gained attention as a potential treatment for various immunological diseases, such as graft-versus-host disease (GvHD) (67), hepatitis C–related vasculitis (68), and type 1 diabetes (69). Low-dose IL-2 promotes a balance between Treg and Th17 cells in patients with Sjögren’s syndrome (SS), a condition characterized by dryness (70). In patients with inflammatory myopathy who received 500,000 IU of IL-2 therapy for 5 days, Treg cell numbers significantly increased, while erythrocyte sedimentation rates, muscle enzyme levels, and pain scores significantly decreased (71). Recent clinical studies have shown that low-dose IL-2 is safe and effective against 11 autoimmune diseases, including rheumatoid arthritis (RA) and ankylosing spondylitis (72). Overall, the IL-2 signaling pathway moderates the role of Tregs in immune regulation and self-tolerance by affecting their development, proliferation, survival, and function.

### 2.3 IL-10

IL-10 is produced by macrophages, monocytes, and T cells and is considered a Th2 cytokine and an anti-inflammatory cytokine. IL-10 signaling requires the presence of cell surface–expressed IL-10 receptors (IL-10R) (73). IL-10 induces STAT3 signaling by phosphorylating the cytoplasmic tails of IL-10R1 and IL-10R2 through Janus-family tyrosine kinase 1 (JAK1) and non-receptor tyrosine-protein kinase (Tyk2), respectively (74). IL-10^−/−^ mice exhibit more severe inflammatory damage, as IL-10 can regulate the function of Tregs through the activation of STAT3, and the lack of IL-10 leads to a significant decrease in STAT3 phosphorylation in microglia. Although the IL-10 promoter lacks binding sites for Foxp3, it does contain binding sites for STAT3, suggesting that Foxp3 may modulate IL-10 expression indirectly through STAT3 signaling. Indeed, Foxp3 is involved in the transcriptional activation of IL-10 by acetylating STAT3 through histone acetyltransferases (HATs), forming the most important connection between IL-10 and Foxp3 (**Figure 1**) (75). Although IL-10 signaling is not required for the induction of Tregs, it is required for Tregs to mediate their suppressive function. For example, IL-10R is indispensable for Tregs to suppress the autoreactive Th17 response (PMID: 21511185). IL-10 may auto-regulate its expression through a negative feedback loop, which involves the autocrine stimulation of IL-10R and inhibition of the p38 signaling pathway (76). Additionally, IL-10 expression is widely regulated at the post-transcriptional level, possibly involving AU-rich element (77), let-7 (78), or miR-106 (79). Under normal conditions, human iTreg cells produce low levels of IL-10. Inhibiting glycogen synthase kinase-3 (GSK3) can significantly upregulate IL-10 expression in Tregs and promote the generation of IL-10^+^ Foxp3^+^ iTreg cells (80).

IL-10 is predominantly secreted by type 1 regulatory T cells (Tr1 cells). Tr1 cells are typically induced from naïve T cells in the periphery, their differentiation can be driven by several factors, including IL-10, IL-27, and TGF-β (81). The transcription factors (TFs) *c-Maf* interacts with *AhR* to synergistically transactivate the IL-10 and IL-21 promoters, thereby promoting IL-27-induced differentiation of murine Tr1 cells (82). Tr1 cells are characterized by their lack of Foxp3 expression and are identified by the co-expression of CD49b and LAG-3, which serve as distinctive markers in both humans and mice (83). Tr1 cells play a critical immunoregulatory role in promoting tolerance in transplant scenarios, such as renal and pancreatic islet transplantation in humans and mice, and in reducing GvHD following hematopoietic stem cell transplantation, largely through their IL-10 mediated activities and antigen-specific actions (84–86). Tr1 cells have also been shown to ameliorate autoimmune diseases by inhibiting pathogenic Th17 response in experimental autoimmune encephalomyelitis and experimental autoimmune uveitis (87, 88).

IL-10 is a major cytokine involved in Treg-mediated immune regulation and immunosuppression (89). It primarily acts on monocytes and macrophages. IL-10 can inhibit the secretion of the pro-inflammatory cytokines TNF-α and IL-1β by monocytes and macrophages (90). IL-10 also inhibits IL-12 synthesis, hindering the differentiation of Th1 cells (73, 91). Blocking the Treg-mediated suppression of Teffs can be achieved by using anti–IL-10 neutralizing antibodies (92). Treg cells regulate the expression of IL-10 through the transcription factor B-lymphocyte-induced maturation protein-1 (Blimp-1). Mice lacking Blimp-1 in peripheral effector CD4^+^ and CD8^+^ T cells show increased cell numbers, while the overexpression of Blimp-1 in T cells promotes the differentiation of Treg cells and enhances their inhibitory effect on T cell proliferation (93). This highlights the importance of IL-10 in mediating the immunosuppressive function of Treg cells.

In addition, IL-10 has various other regulatory functions. It can induce the differentiation of Th0 cells into helper T cells (Th2), while inhibiting Th1 differentiation, thereby affecting the balance between Th1 and Th2 immune responses (94). IL-10 can also inhibit antigen presentation and prevent monocytes and macrophages from producing pro-inflammatory cytokines. IL-10 can inhibit the secretion of IL-6 and IL-12 by dendritic cells (DCs), thereby suppressing Th17 differentiation (95–97). Compared to wild-type mice, IL-10^−/−^ mice exhibit more severe arthritis, decreased numbers of Tregs, decreased expression of Foxp3, and increased numbers of Th1 and Th17 cells. This further confirms that IL-10 may work in coordination with Tregs and other immune cells to inhibit the differentiation and development of Th1 and Th17 cells, exerting negative immune regulatory effects (98). The immunostimulatory capacity of IL-10 in the context of immunoregulation has been demonstrated. IL-10 expression in tumor cell lines transfected from IL-10 transgenic mice controls primary tumor growth and reduces the burden of metastasis (99). Recombinant mouse IL-10 has been shown to induce IFN-γ and CD8^+^ T cell–dependent anti-tumor immunity *in vivo* (100, 101).

### 2.4 IL-35

IL-35, a member of the IL-12 family, is a heterodimeric protein that consists of p35 and Epstein-barr virus-induced gene 3 (EBI3) (102). It can be secreted by Tregs. IL-35 can be expressed in various tissues and environments, such as the thymus, peripheral lymphoid organs, and inflammatory sites, and influences the generation and maintenance of Tregs by activating the IL-35 receptor (IL-35R) (103). IL-35 not only suppresses effector T cells but also promotes the conversion of CD4^+^ T cells into IL35-producing induced regulatory T cells (iTr35). IL-35 triggers signal transduction by binding to IL-35R, which is composed of two subunits (i.e. IL-12Rβ2 and IL-27Rα) and subsequently activates the JAK family (102). Specifically, IL-35 induces the phosphorylation and activation of the JAK1 and Tyk2 kinases (104). Activated JAK further phosphorylates and activates STAT proteins, including STAT1 and STAT4 (**Figure 1**) (105). These phosphorylated STAT proteins undergo conformational changes, form dimers or multimers, and then translocate to the cell nucleus, where they can activate the transcription of the Foxp3.

The function of Tregs proved effective in EBI3^−/−^ and p35^−/−^ mice, that is, IL-35 subunit knockout models, suggesting that IL-35 plays an important role in maintaining the function of Tregs (106). The immunosuppressive capacity of Tregs in EBI3^−/−^ and p35^−/−^ mice was significantly diminished compared to that of Tregs in wild-type mice. In cases of human colon cancer, the expression level of IL-35 in tumor tissues was positively correlated with the degree of malignancy and clinical stage of the tumor. Additionally, a strong positive correlation between the level of IL-35 expression and the number of Tregs in peripheral blood has been noted (107). Therefore, tumor-derived IL-35 may promote tumor growth by recruiting Tregs into the TME (108). Another study conducted by Meghan et al. demonstrated that targeting IL-35 can potentially serve as a therapeutic strategy for tumor suppression. Their research findings revealed that the neutralization of IL-35 led to enhanced tumor control in wild-type C57BL/6 mice when compared to control mice (109).

In summary, IL-35 plays an important role in immune regulation and immune balance by regulating the development of Tregs, enhancing Foxp3 expression and function, and exerting immunosuppressive effects.

## 3 Treg-inhibitory cytokines

### 3.1 IL-6

IL-6 is an inflammatory cytokine linked to autoimmune and inflammatory diseases (110). It can be produced by lymphoid and some non-lymphoid cells. It can also be secreted by fibroblasts, endothelial cells, keratinocytes, mesangial cells, and tumor cells. The pro-inflammatory properties of IL-6 include inhibition of the immunosuppressive capacity of Tregs and interference with their differentiation from naïve T cells (111). Studies have shown that high levels of IL-6 and IFN-γ inhibited the expression of Foxp3 during the differentiation of Tregs (112, 113). In early pregnancy, C57BL/6 models, which are susceptible to congenital toxoplasmosis, exhibited elevated IL-6 but reduced expression of Foxp3 in response to congenital toxoplasmosis infection when compared to the infection-resistant Balb/c models (114). *In vitro* activation of purified mouse CD4^+^ CD25^+^ Foxp3^+^ T cells caused their differentiation into Th17 in the presence of IL-6 (47, 115, 116). Therefore, IL-6 is the key factor determining whether naïve CD4^+^ T cells differentiate into Treg or Th17 cells (117). The deletion of IL-6 and TGF-β in mice contributes to the depletion of Th17 cells, which leads to the failure of EAE modeling (118–120). When IL-6 is present at inflammatory sites, such as sites of mucosal inflammation, it can cause phosphorylation of the downstream STAT3 through tyrosine (121). Hyperactivity of p-STAT3 can increase the expression of the transcription factor RORγt in T cells, followed by a decrease in Foxp3 expression, thus causing CD4^+^ T cells to differentiate into Th17 cells instead of Tregs (120, 122). Another study reported that when co-cultured with multiple myeloma (MM) cells, bone marrow stromal cells could secrete IL-6 and thereby transform Tregs into Th17 cells, a finding further verified in animal models (123, 124). In preclinical studies, IL-6 monoclonal antibodies demonstrated positive drug synergies (e.g., between bortezomib, melphalan, and dexamethasone), thereby enhancing the effectiveness of MM treatment. This improvement may be attributed to the Treg/Th17 ratio (125, 126). Interestingly, retinoic acid, a metabolite of vitamin A, could regulate TGF-β–dependent immune responses and prevent IL-6 from inducing pro-inflammatory Th17 cells and promoting the differentiation of anti-inflammatory Tregs (127), indicating the complexity of the balance of Th17 cells and Tregs.

### 3.2 TNF-α

TNF-α is an inflammatory cytokine that mediates inflammation and may cause tissue damage. It is secreted by macrophages, monocytes, neutrophils, CD4^+^ T cells, and natural killer (NK) cells. While TNF-α is typically known for enhancing immune responses and promoting inflammation, it has also been shown to inhibit Treg function (128). TNF-α suppresses the differentiation and development of Tregs, leading to a reduction in their numbers (129). During Treg differentiation, the presence of TNF-α interferes with the TGF-β signaling pathways. This involves disruption of the activation and transduction of downstream signaling molecules, such as the phosphorylation and nuclear translocation of the SMAD protein family, subsequently affecting Foxp3 expression and Treg differentiation (129). Previous studies have reported that TNF-α, through tumor necrosis factor receptor 2 (TNFR2) activation, played a role in the expansion and amplification of Tregs (130). Approximately 30–40% of peripheral blood Tregs express TNFR2, which can be upregulated by TNF-α (131). Interestingly, only Tregs that express TNFR2 exhibit strong immunosuppressive activity, while Tregs lacking TNFR2 display minimal to no immunosuppressive activity. Therefore, the TNF–TNFR2 signaling pathway is necessary for maintaining the function and phenotypic stability of Tregs in the body (132). When compared to conventional CD4 single-positive cells, members of the TNF receptor superfamily, including glucocorticoid-induced TNFR-related (GITR), CD134, and TNFR2, are overexpressed on Treg precursor cells. These receptors enhance T cell receptor (TCR) signaling through TGF-β–activated kinase 1 (TAK1) and CD28-dependent signaling pathways. Treg precursor cells lacking TAK1 and CD28 cannot express GITR, CD134, or TNFR2, resulting in the inhibition of Foxp3^+^ Treg maturation (133).

The systemic injection of an anti–TNF-α neutralizing antibody, such as infliximab, can induce IL-10 in CD4^+^ T cells and Th17 cells, as well as Aiolos binding of conserved regions of IL-10. However, in the treatment of Crohn’s disease with anti–TNF-α therapy, IL-17^+^ cells in the intestines of patients decreased significantly after 3 months of treatment, and Foxp3^+^ cells were unstable. However, the IL-17^+^/CD4^+^ and IL-17^+^/Foxp3^+^ ratios were both decreased, suggesting that TNF-α and a balanced relationship between Th17 cells and Tregs are key factors in the treatment of the disease (134–136). TNF-α can damage the function of T cells by enhancing the dephosphorylation of Foxp3; and their function can be restored by TNF-α antagonist therapy, thereby indirectly regulating the interaction between T cells and Th17 and Th1 cells, which affects autoimmune inflammation in RA (137). It is important to note that the inhibitory effects of TNF-α on Tregs may be beneficial in certain contexts, such as enhancing anti-tumor immune responses by reducing the suppressive effects of Tregs (138). The accumulation of Tregs in the TME has been identified as one of the major factors in the initiation and development of immune checkpoint inhibitor resistance (139). The CC motif chemokine receptor 8 (CCR8) is a marker of activated inhibitory Tregs and has a significant impact on the function of Tregs in the TME (140). High levels of TNF-α in the colorectal cancer (CRC) TME upregulate CCR8 expression in Tregs via the TNFR2/NF-κB signaling pathway and Foxp3 transcription factor. Depletion or blockade of TNFR2 inhibits gastrointestinal tumor progression by reducing CCR8^+^ Treg infiltration, thereby enhancing the efficacy of anti–PD-1 therapy (140, 141).

## 4 Co-stimulatory molecules

**Figure 2:**
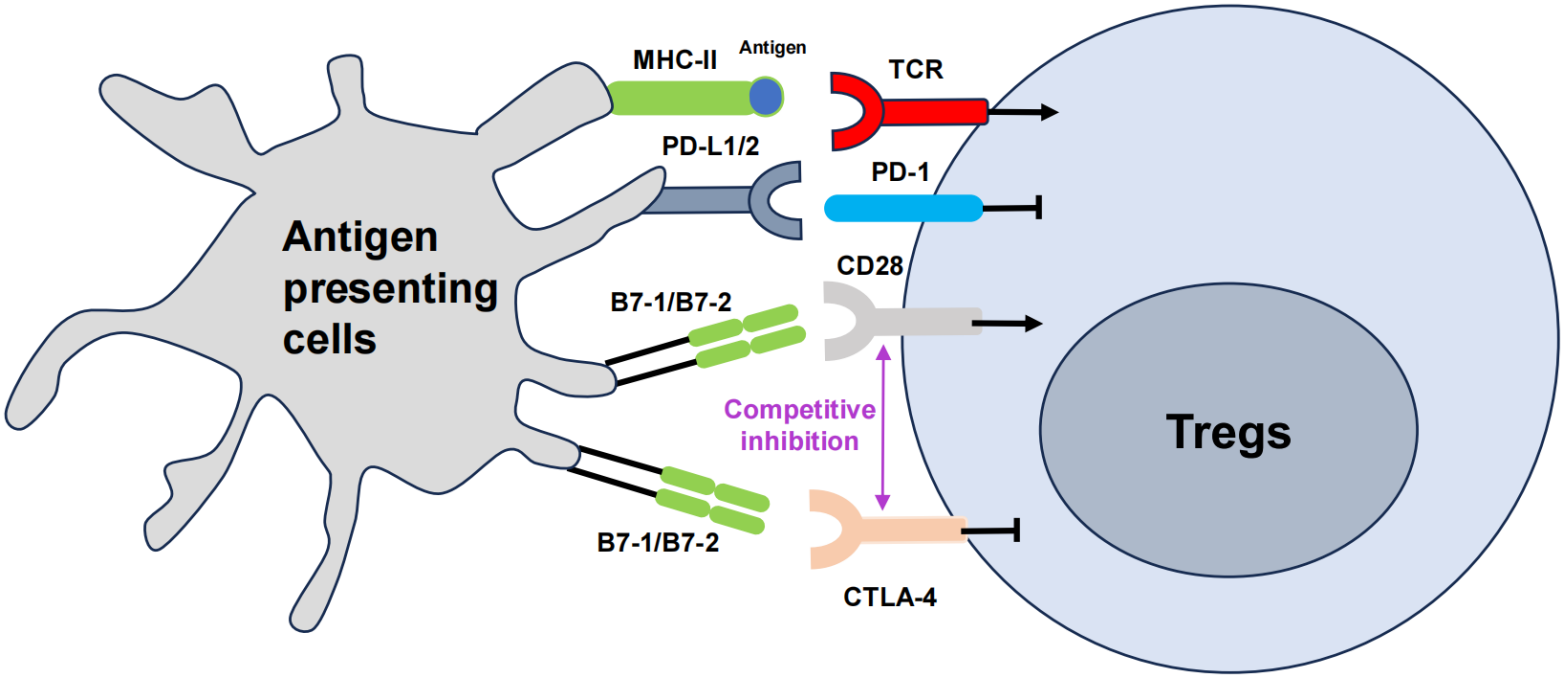
The role of co-stimulatory molecules in Treg cells, namely CTLA-4 and PD-1. The T cell receptor (TCR) engages the major histocompatibility complex (MHC)-peptide complex on APCs. The interaction between CD28 on T cells and B7 on APCs triggers costimulatory signaling, which is vital for T cell activation. Cytotoxic T lymphocyte-associated antigen 4 (CTLA-4) mitigates this activation by outcompeting CD28 for its ligands B7, thus attenuating the costimulatory signal. Concurrently, the interaction of programmed cell death protein 1 (PD-1) on T cells with its ligands PD-L1 or PD-L2 also transmitted by APCs, further modulates immune responses, generally by dampening T cell activity. The arrows represent positive regulation of the Tregs response, and the horizontal lines represent negative regulation.

Co-stimulatory molecules, such as CTLA-4 and PD-1 are also crucial for Treg cell activation and function (**Figure 2**). The B7 family is composed of a group of cell-surface molecules primarily found on antigen-presenting cells (APCs), such as DCs, macrophages, and B cells (142). These molecules provide co-stimulatory or co-inhibitory signals that are crucial for the activation, differentiation, and survival of T cells, thereby playing an essential role in the regulation of T cell–mediated immune responses, especially in the field of immuno-oncology (143). CTLA-4, PD-1, and PD-L1 are the most extensively studied and clinically applied immune checkpoint molecules to date (144). Some clinical trials and studies have shown that the combined use of nivolumab (a PD-1 inhibitor) and ipilimumab (a CTLA-4 blocker) is more effective for patients with advanced melanoma (145). The use of immune checkpoints has ushered in a new era in tumor treatment.

### 4.1 CTLA-4

CTLA-4 is a co-stimulatory molecule expressed on the surface of effector T cells (146). Interestingly, Treg cells also constitutively express CTLA-4 for fine tuning T cell activation through the obstruction of co-stimulatory signals (147). CTLA-4 exhibits higher binding affinity for the co-stimulatory molecules CD80/CD86 than CD28, thereby effectively outcompeting it (148). Additionally, CTLA-4 is involved in the ‘trans-endocytosis’ of CD80 and CD86 from APCs, further inhibiting their availability for co-stimulatory interactions (147). These actions are crucial for Treg cells to exert control over T cell activation and to prevent autoimmune responses (149). The absence of CTLA-4 disrupts Treg cell function, leading to unchecked proliferation and activation of Tconvs, which can result in autoimmune pathology (150). Researchers have shown that introducing the extracellular domain of CTLA-4 (cdCTLA-4) into mice lacking CTLA-4 fully restores Treg activity, suggesting that cdCTLA-4 is sufficient to provide inhibitory function (151). This implies that CTLA-4 function may not necessarily involve a signal transduction process. In another study, the expression of CTLA-4 in CD4^+^ CD25^+^ Foxp3^+^ Treg cells was elevated both in the blood of patients with pulmonary tuberculosis and in the pleural cavity of individuals with tuberculosis pleurisy. Blocking CTLA-4 weakened the ability of Foxp3^+^ Tregs to suppress the IFN-γ T effector response to the effect of purified protein derivative (PPD) stimulation, and this reversal effect was not consistent with the decrease in IL-10. Blocking CTLA-4 reversed the ability of Tregs to inhibit PPD-driven IFN-γ and IL-2 responses at the mRNA level, while IL-10 and TGF-β did not show significant changes. Blocking CTLA-4 significantly eliminated the inhibitory effect of Foxp3^+^ Tregs on the PPD-specific T cell proliferation response (152). Therefore, CTLA-4 is a promising new target for immunotherapy for active tuberculosis (153).

Research on CTLA-4 is still in the stage of determining its physical activity. However, although the mechanism is currently obscure, it does not affect the positive clinical effects of anti–CTLA-4 drugs in arousing an immune response and treating tumorigenicity. CTLA-4-targeting agents serve various immunomodulatory roles. Abatacept (Orencia), which includes the CTLA-4 domain, is employed for treating RA and is used to prevent organ transplant rejection, as seen with belatacept (Nulojix). Distinct from ipilimumab (Yervoy), an FDA-approved melanoma treatment that blocks CTLA-4 to stimulate the immune response, abatacept mimics CTLA-4 on T cells. It competes with CD28 for binding to B7 molecules on APCs, thereby blocking the co-stimulatory signal required for T cell activation (154). Belatacept, a derivative of abatacept, with an alteration of merely two amino acids, exhibits a tenfold increase in activity compared to its precursor, more effectively inhibiting the CD28-mediated co-stimulatory signaling of T cells (155). The idea of CTLA-4 target druggability is mainly based on the high-affinity binding of anti-CTLA-4 antibodies to CTLA-4 molecules, mediating Treg depletion or functional blockade, thereby enhancing T cell activation and the immune response to cancer (156). CTLA-4 is involved in maintaining tolerance to autoimmune diseases, such as diabetes, as well as spontaneous abortion tendencies (157–159). Single nucleotide polymorphisms in exon 1 of CTLA-4 have been linked to susceptibility to several autoimmune diseases, including multiple sclerosis (160). The antibody-mediated blocking of CTLA-4 prevents the development of tolerance, enhances the anti-tumor response, and exacerbates autoimmune disease (161). In clinical trials, CTLA-4 development is mainly divided into two treatment modalities: monoclonal antibody or combined with PD-1/PD-L1 monoclonal antibody. Bispecific antibodies have also been constructed by association with other popular targets (162).

### 4.2 PD-1

PD-1 is a co-inhibitory receptor primarily expressed on activated T cells, B cells, and APCs. PD-1, through binding to its ligands PD-L1 and PD-L2, inhibits T cell activation and function to prevent excessive immune responses (163). Tregs express higher levels of PD-1 on their surfaces compared to CD4^+^ Th cells (164). This indicates that PD-1 plays an important role in the regulation of Tregs. When PD-1 binds to its ligands, PD-L1 or PD-L2, it exerts inhibitory effects (163). PD-1 activation suppresses signal transduction pathways that activate T cells, leading to reduced cell proliferation and cytokine production, thereby limiting the intensity and duration of immune responses (165). The PD-1 signaling pathway in Tregs can regulate their suppressive function and immune regulatory roles. Evidence from multiple studies indicates that PD-1 inhibits the suppressive abilities of Tregs (166). Isolation of PD-1^hi^ and PD-1^−^ cells from the peripheral blood of healthy individuals has revealed that Tregs with higher levels of PD-1 show diminished suppressive function and elevate production of IFN-γ (167). Mouse model experiments further demonstrate that Tregs lacking PD-1, or those from mice treated with PD-1 blocking antibodies, exhibit enhanced suppressive capabilities (168). In tumor environments, blocking PD-1 not only improves the function of PD-1^+^ CD8^+^ T cells but also intensifies the immunosuppressive effects of PD-1^+^ Tregs (169). As a result, the effectiveness of PD-1 inhibitors in treating patients is determined by their complex interplay with both effector T cells and Tregs, which highlights the crucial role of the PD-1 in controlling Tregs.

An *in vivo* mouse MC38 (CRC cell line) subcutaneous transplantation tumor model and an azoxymethane (AOM)/dextran sodium sulfate (DSS)–induced spontaneous CRC model both confirmed that gallic acid can affect Foxp3 protein levels by targeting the expression of ubiquitin specific peptidase 21 (Usp21) in Tregs, inducing the formation of Th1-like Tregs and reducing their immunosuppressive function (170). Simultaneously, gallic acid can enhance the anti-tumor effect of anti–PD-1 immune checkpoint blocking and downregulate the expression of PD-L1 protein in Tregs, indicating that the deubiquitinating enzyme Usp21 can deubiquitinate and stabilize the PD-L1 protein. PD-1 was also expressed on CD4^+^ Foxp3^+^ CXCR5^−^ Tregs and inhibited the activity of lymphocytes by downregulating then maintaining the expression level of Foxp3 protein (171). Neuropilin-1 (NRP-1) plays an essential role in maintaining the stability and function of Tregs within tumors (172). On the one hand, an increase in the NRP-1 phenotype induces the production of IFN-γ in the tumor, which increases the vulnerability of Tregs, weakens tumor immunosuppression, and enhances antitumor immunity, which is related to the prognosis of melanoma (173) and head and neck squamous cell carcinoma (174). On the other hand, the susceptibility of Treg cells to IFN-γ, which is induced by anti–PD-1 therapy, is a potential mechanism underlying the effectiveness of anti–PD-1 drugs. These drugs stimulate the production of significant quantities of IFN-γ (175). Recently, several studies have reported correlations between tumor PD-L1 expression, objective response rate, and PD-1/PD-L1 inhibitors, suggesting that PD-L1 may become an effective biomarker (176, 177).

## 5 Potential of Treg-based immunotherapy

The overall outcome of these interactions between Treg subpopulations, cytokines, and co-stimulatory molecules is a finely-tuned immune system that can respond to pathogens aggressively. Based on the characteristics of different subsets of Tregs, different strategies can be utilized to treat autoimmune diseases (178, 179). To date, early clinical trials using Treg cell therapy have shown great promise in the fields of transplantation rejection (180), GvHD (181), and autoimmune diseases (179). However, one of the main challenges in these studies is the isolation of pure Tregs and their expansion to a sufficient clinical dose. The principle of Treg cell therapy is to restore the balance between Teffs and immune regulatory cells by injecting an effective dose of Tregs into the patient’s body, thus promoting immunological tolerance (182).

### 5.1 Autoimmune diseases

Tregs play a pivotal role in upholding immune tolerance to self-antigens, thereby preventing the activation and proliferation of self-reactive T cells that may not be eliminated during thymic selection. They also inhibit APCs, such as DCs, which are instrumental in triggering immune responses (183). Furthermore, Tregs interact with other immune cells, including B cells and NK cells, establishing a balanced immune system (184).

Autoimmune diseases include systemic autoimmune diseases and organ-specific autoimmune diseases. Representative diseases of the former type include systemic lupus erythematosus, SS, and RA (185), and the latter include, for example, type 1 diabetes (186), pemphigus (187), and Hashimoto thyroiditis (188). Treg therapy can alleviate arthritis symptoms by suppressing inflammatory responses and regulating the immune system. In addition, It can regulate intestinal immune balance and reduce intestinal inflammation and tissue damage, thus alleviating inflammatory bowel diseases (such as Crohn’s disease and ulcerative colitis) (189, 190). Treg therapy can also alleviate autoimmune hepatitis and improve liver function (191) and systemic lupus erythematosus (192). Finally, Treg therapy can be used for other autoimmune diseases, such as multiple sclerosis (193), myasthenia gravis (194), and autoimmune thyroid diseases (195).

Our recent studies revealed that the intravitreal injection of Tregs resolved ocular inflammation in experimental autoimmune uveitis (116). In patients with RA, synovial Tregs lose their suppressive functions. They fail to inhibit the production of pro-inflammatory cytokines, such as TNF-α and IFN-γ, by other CD4^+^ T cells and monocytes and to inhibit the proliferation of Teffs (196). In animal models, the adoptive transfer of Tregs significantly reduces disease severity, highlighting the importance of Tregs in controlling abnormal joint inflammatory responses (197). Tregs can also suppress the activity of other immune cells through immunoregulatory molecules, for example, TGF-β and IL–10 produced by Tr1 cells, to inhibit inflammatory reactions and reduce self-attack on joint tissues, thereby alleviating symptoms of RA (198). Other autoimmune diseases, such as inflammatory bowel disease (199), systemic lupus erythematosus (200), and autoimmune thyroid diseases (201), including thyroid nodules, thyroiditis, Graves’ disease, and autoimmune hypothyroidism, can all be alleviated by enhancing the immunosuppressive function of Tregs. Today, adoptive Treg therapy has been widely used and tested in autoimmune diseases (**Table 2**).

**Table 2:** Animal models of autoimmune disease and inflammation.

Disease	Type	Treg-based immunotherapeutic effects	Influence
Connective tissue autoimmune disease	Systemic lupus erythematosus (202)	Activated Tregs	IFN-γ↓ IL-17↑
	Rheumatoid arthritis (203)	CD4^+^ CD25^−^ T & mature tolerant DCs promote CD4^+^ CD25^+^ Tregs	TNF-α↓ IL-17↓ IL-6↓ IFN-γ↑ IL-10↑ TGF-β↑
Neuromuscular autoimmune disease	Experimental autoimmune encephalomyelitis (204)	CCL1–Ig promotes CCR8^+^ Tregs	CD39↑ GranB↑ IL-10↑
	Experimental autoimmune myasthenia gravis (205)	EAMG CD4^+^ & marrow DCs promote DC EAMG Tregs	Clinical score↓ AChR↓
Endocrine autoimmune disease	Type 1 diabetes (206)	Antigen-specific Tregs	IL-10↑ TGF-β↑ CD8^+^ T↓ CD8^+^/CD4^+^ T↓
	Premature ovarian insufficiency (207)	Activated Tregs	Follicle-stimulating hormone↓ Luteinizing hormone↓ Anti-zona pellucida antibody↓ Estradiol↑ Anti-Müllerian hormone↑
Autoimmune diseases of the digestive system	Autoimmune hepatitis (208)	CD4^+^ CD25^+^ Tregs & HSCs promote HSC Tregs	AST↓ ALT↓ Treg/Th17 -
	Ulcerative colitis (209)	Activated Tregs	IL-1↓ TNF-α↓ NO↓ PGE2↓
Other autoimmune diseases	Graft-versus-host disease (210)	Tr1 cells promote Tregs	Th2/Th1↑ Treg/Th17↑

### 5.2 Transplantation

In transplantation, Tregs are critical for promoting graft tolerance and reducing transplant rejection rates. In a study of immune rejection therapy for kidney transplantation, 11 patients were followed for 60 weeks after transplantation to assess immune response, rejection, and renal function. Of these patients, eight were successfully maintained on monotherapy immunosuppression. Additionally, 10 patients who received Tregs treatment could be weaned off immunosuppression to low dose tacrolimus monotherapy within 48 weeks, although eight patients later experienced failure of tacrolimus monotherapy. Despite the need for additional immunosuppressive treatments, all 11 patients in the trial maintained good graft function at the 3-year follow-up time point. The study’s authors successfully developed a method for isolating and expanding autologous polyclonal Tregs from a small blood sample and demonstrated the feasibility of this treatment (211).

Graft rejection reactions may occur after bone marrow transplantation, leading to transplant failure. Treg therapy can regulate the immune response after transplantation, reducing the occurrence of graft rejection (212). Amarnath et al. (213) found that Tregs could promote the generation of bone marrow DCs and reduce their ability to stimulate the generation of efficient T cells in GvHD. Tregs have high surface expression levels of PD-L1, which can bind to PD-L1 of DCs, thus inhibiting the activation of T cells and alleviating GvHD (214, 215). In addition, Tregs can express inhibitory molecules (e.g. CTLA-4, LAG-3, and NRP-1) to inhibit T cell activation (216). Tregs can also express CD62L and CCR5, maintain *in vivo* homing characteristics, inhibit the activation of early Teffs, and induce transplantation immune tolerance (217). The development of CAR-T technology has promoted the development of clinical trials using CAR Tregs to treat transplantation immune rejection (218). HLA-A2 is the main molecular target that causes immune rejection (219). CAR HLA-A2 Tregs can significantly reduce the inflammation and rejection caused by grafts, can promote the immune tolerance of grafts, and are superior to polyclonal Tregs in preventing GvHD caused by donor T cells (220). CAR Treg cell therapy likely has the advantage of avoiding transplant rejection. Early treatment may help prevent the occurrence of rejection reactions, while later treatment may be more suitable for the management of existing rejection reactions.

### 5.3 Cancer treatment

Tregs, although vital for preserving immune tolerance and preventing autoimmune diseases, can be counterproductive in cancer by inhibiting anti-tumor responses and enabling cancer cells to escape immune surveillance. Consequently, restricting Treg activity is essential for enhancing the immune system’s ability to combat cancer. Targeting Tregs also has potential applications in cancer treatment. The goal of targeting Tregs for cancer treatment is to enhance the tumor immune response and suppress tumor growth by modulating the activity of the immune system (221). Immune checkpoint inhibitors have become an important strategy in cancer treatment (222). In this treatment approach, the suppressive effects of Tregs may inhibit the efficacy of immune checkpoint inhibitors. Therefore, reducing or modulating the immunosuppressive effects of Tregs can enhance the efficacy of immune checkpoint inhibitors (182). CpG combined with low-dose anti-OX40/CTLA-4 triple immunotherapy can eliminate Tregs in a tumor and has a curative effect on central nervous system lymphoma in mice (223). In patients with tumors that did not respond to PD-1 monotherapy, the combination of the partial deletion of CARD-containing MAGUK protein 1 (CARMA1) and the sterol regulatory element binding protein (SREBP) inhibitor fatostatin produced a strong antitumor effect (224). This is because, upon the destruction of the CARMA1–BCL10–MALT1 semaphore complex, most tumor-infiltrating Tregs inhibit IFN-γ derived from CD8^+^ T cells, which inhibits the growth of immunosuppressive M2-like tumor-associated macrophages (TAMs) (225). Fatostatin inhibits SREBP1-mediated fatty acid synthesis, inhibits the occurrence and development of TAMs, and then controls tumor growth.

Chemokine pathway blocking and specific target blocking have also been used to suppress tumors (**Table 3**) (226). Blocking the migration of Tregs to the TME is a new direction of tumor immunotherapy (227). Tregs in a canine bladder cancer model entered tumor tissue through the CCL17–CCR4 axis, and anti-CCR4 treatment significantly inhibited tumor growth and improved the survival rate. In addition, CCR4 was highly expressed in tumor-infiltrating Tregs (TITRs) in human bladder cancer (228). Another study showed that the number of TITRs in CD36^−/−^ Treg mice was decreased, the anti-tumor activity of tumor-infiltrating lymphocytes was enhanced, and tumor growth was inhibited (229). Neuropilin 1 (Nrp1)^−/−^ Tregs in mice with a partial Nrp1 knockout can prevent wild-type (Nrp1^+/+^) Tregs from performing their immunosuppressive function by secreting IFN-γ, thus promoting the clearance of melanoma (10). New drugs are continuously being developed to inhibit cancers. Some of the difficulties of tumor immunotherapy include eliminating the immunosuppressive effect of the TME and enhancing the specific anti-tumor response. Further effective differentiation between TITRs and tissue-resident Treg phenotypes or transcription levels, as well as a continuous reduction in the dynamic differences in Tregs between preclinical models and patient-derived samples, can provide more therapeutic bases for immunotherapy based on Treg targets.

**Table 3:** Methods of Treg-targeted therapy for tumors and their corresponding cytokines.

No.	Therapeutic effect	Drug	Target
1	Treg depletion in the TME	Kinase inhibitor, cyclophosphamide, anti-CD25	IL-2 signaling
2	Halting Treg migration	Anti-CCR4, anti-CCR8	CCR4, CCR8
3	Sensitizing intertumoral Tregs to checkpoint blockade	Anti-TIGIT, anti–LAG-3, LAG-3–Ig fusion protein, nonfucosylated anti–CTLA-4	LAG-3, TIM3, TIGIT, CTLA-4
4	Targeting the co-stimulation of Tregs	GITR agonist, OX40 agonist, ICOS agonist and antagonist, TNFR2 antagonist	GITR, ICOS, OX40, TNFR2, NRP-1
5	Targeting Treg cytokines	Anti–IL-10, anti-GARP, anti–IL-35	IL-10, TGF-β, IL-35
6	Altering Treg fragility	PI3Kδ inhibitor, anti–NRP-1	NRP-1, IFN-γ
7	Targeting Treg metabolism	Meformin, IDO inhibitor, A2AR inhibitor, Orencia, Nulojix	IL-10, CTLA-4, FOXP3

*IL, Interleukin; CD, Cluster of differentiation; CCR, Hemokine (C-C motif) receptor; LAG, Lymphocyte activation gene; TIM, T cell immunoglobulin domain and mucin domain; TIGIT, T cell immunoreceptor with Ig and immunoreceptor tyrosine-based inhibitory motif (ITIM) domains; CTLA, Cytotoxic T lymphocyte-associated antigen; GITR, Glucocorticoid-induced tumor necrosis factor receptor; ICOS, Inducible synergistic co-stimulation molecules; OX40, CD134 & TNF receptor superfamily member 4 (TNFRSF4); TNFR2, Tumor necrosis factor receptor; NRP, Neuropilin; TGF, Transforming growth factor; IFN, Interferon; FOX, Forkhead box protein.

The exploration and deployment of therapeutics targeting Tregs are extensive, chiefly because these cells express an array of receptors on their surface. The most utilized are agonists of the TNFR superfamily and antagonists of immune checkpoint inhibitors (230, 231). These modulators alleviate autoimmune conditions and bolster anti-tumor responses by influencing Treg function. Next-generation Treg interventions focus on directing Tregs to selectively recognize tissue or organ-specific antigens by incorporating a chimeric antigen receptor (CAR) structure. Engineered CAR-Tregs are also being designed to convert pro-inflammatory cytokine signals into those of IL-2 or IL-10, which intensifies the suppression of inflammation (232).

## 6 Conclusion

The study of Treg signaling pathways provides a theoretical basis for immune balance and the treatment of autoimmune diseases. By modulating the quantity and function of Tregs, immune system activity can be balanced, inflammation can be alleviated, and the development and progression of autoimmune diseases can be prevented and treated. Preliminary results from clinical trials demonstrate the potential of Treg therapy in the fields of transplant rejection, autoimmune diseases, and cancer. Although the application of Treg therapy poses challenges, personalized treatment strategies and the optimization and monitoring of treatment processes will contribute to improving its safety and efficacy and promoting its further clinical implementation.

## Conflicts of Interest

The authors declare that the research was conducted in the absence of any commercial or financial relationships that could be construed as a potential conflict of interest.

## Author contributions

ZY: Writing – original draft, Writing – review & editing. KD: Writing – original draft, Investigation, Software. WC: Writing – original draft, Writing – review & editing.

## References

[1] Sakaguchi S Sakaguchi N Asano M Itoh M Toda M. Immunologic self-tolerance maintained by activated T cells expressing il-2 receptor alpha-chains (Cd25). Breakdown of a single mechanism of self-tolerance causes various autoimmune diseases. J Immunol. (1995) 155:1151–64. 7636184

[2] Boehm F Martin M Kesselring R Schiechl G Geissler EK Schlitt HJ. Deletion of foxp3+ Regulatory T cells in genetically targeted mice supports development of intestinal inflammation. BMC Gastroenterol. (2012) 12:97. doi: 10.1186/1471-230X-12-97 22849659 PMC3449180

[3] Collison LW Pillai MR Chaturvedi V Vignali DA. Regulatory T cell suppression is potentiated by target T cells in a cell contact, il-35- and il-10-dependent manner. J Immunol. (2009) 182:6121–8. doi: 10.4049/jimmunol.0803646 PMC2698997 19414764

[4] Nakamura K Kitani A Strober W. Cell contact-dependent immunosuppression by cd4(+)Cd25(+) regulatory T cells is mediated by cell surface-bound transforming growth factor beta. J Exp Med. (2001) 194:629–44. doi: 10.1084/jem.194.5.629 PMC2195935 11535631

[5] Pandiyan P Zheng L Ishihara S Reed J Lenardo MJ. Cd4+Cd25+Foxp3+ Regulatory T cells induce cytokine deprivation-mediated apoptosis of effector cd4+ T cells. Nat Immunol. (2007) 8:1353–62. doi: 10.1038/ni1536 17982458

[6] Tekguc M Wing JB Osaki M Long J Sakaguchi S. Treg-expressed ctla-4 depletes cd80/cd86 by trogocytosis, releasing free pd-L1 on antigen-presenting cells. Proc Natl Acad Sci U.S.A. (2021) 118:e2023739118. doi: 10.1073/pnas.2023739118 34301886 PMC8325248

[7] Kazanova A Rudd CE. Programmed cell death 1 ligand (Pd-L1) on T cells generates treg suppression from memory. PloS Biol. (2021) 19:e3001272. doi: 10.1371/journal.pbio.3001272 34010274 PMC8168839

[8] Cross AR Lion J Poussin K Glotz D Mooney N. Inflammation determines the capacity of allogenic endothelial cells to regulate human treg expansion. Front Immunol. (2021) 12:666531. doi: 10.3389/fimmu.2021.666531 34305898 PMC8299527

[9] Zhang L Zhang M Xu J Li S Chen Y Wang W. The role of the programmed cell death protein-1/programmed death-ligand 1 pathway, regulatory T cells and T helper 17 cells in tumor immunity: A narrative review. Ann Transl Med. (2020) 8:1526. doi: 10.21037/atm-20-6719 33313271 PMC7729304

[10] Overacre-Delgoffe AE Chikina M Dadey RE Yano H Brunazzi EA Shayan G. Interferon-gamma drives T(Reg) fragility to promote anti-tumor immunity. Cell. (2017) 169:1130–41 e11. doi: 10.1016/j.cell.2017.05.005 28552348 PMC5509332

[11] Kondelkova K Vokurkova D Krejsek J Borska L Fiala Z Ctirad A. Regulatory T cells (Treg) and their roles in immune system with respect to immunopathological disorders. Acta Med (Hradec Kralove). (2010) 53:73–7. doi: 10.14712/18059694.2016.63 20672742

[12] Rocamora-Reverte L Melzer FL Wurzner R Weinberger B. The complex role of regulatory T cells in immunity and aging. Front Immunol. (2020) 11:616949. doi: 10.3389/fimmu.2020.616949 33584708 PMC7873351

[13] Workman CJ Szymczak-Workman AL Collison LW Pillai MR Vignali DA. The development and function of regulatory T cells. Cell Mol Life Sci. (2009) 66:2603–22. doi: 10.1007/s00018-009-0026-2 PMC2715449 19390784

[14] Russler-Germain EV Rengarajan S Hsieh CS. Antigen-specific regulatory T-cell responses to intestinal microbiota. Mucosal Immunol. (2017) 10:1375–86. doi: 10.1038/mi.2017.65 PMC5939566 28766556

[15] Chen W Jin W Hardegen N Lei KJ Li L Marinos N. Conversion of peripheral cd4+Cd25- naive T cells to cd4+Cd25+ Regulatory T cells by tgf-beta induction of transcription factor foxp3. J Exp Med. (2003) 198:1875–86. doi: 10.1084/jem.20030152 PMC2194145 14676299

[16] Duhen T Duhen R Lanzavecchia A Sallusto F Campbell DJ. Functionally distinct subsets of human foxp3+ Treg cells that phenotypically mirror effector th cells. Blood. (2012) 119:4430–40. doi: 10.1182/blood-2011-11-392324 PMC3362361 22438251

[17] Halim L Romano M McGregor R Correa I Pavlidis P Grageda N. An atlas of human regulatory T helper-like cells reveals features of th2-like tregs that support a tumorigenic environment. Cell Rep. (2017) 20:757–70. doi: 10.1016/j.celrep.2017.06.079 PMC5529316 28723576

[18] Chung Y Tanaka S Chu F Nurieva RI Martinez GJ Rawal S. Follicular regulatory T cells expressing foxp3 and bcl-6 suppress germinal center reactions. Nat Med. (2011) 17:983–8. doi: 10.1038/nm.2426 PMC3151340 21785430

[19] Reinhardt RL Liang HE Locksley RM. Cytokine-secreting follicular T cells shape the antibody repertoire. Nat Immunol. (2009) 10:385–93. doi: 10.1038/ni.1715 PMC2714053 19252490

[20] Fu W Liu X Lin X Feng H Sun L Li S. Deficiency in T follicular regulatory cells promotes autoimmunity. J Exp Med. (2018) 215:815–25. doi: 10.1084/jem.20170901 PMC5839755 29378778

[21] Shevach EM Thornton AM. Ttregs, ptregs, and itregs: similarities and differences. Immunol Rev. (2014) 259:88–102. doi: 10.1111/imr.12160 24712461 PMC3982187

[22] Gottschalk RA Corse E Allison JP. Expression of helios in peripherally induced foxp3+ Regulatory T cells. J Immunol. (2012) 188:976–80. doi: 10.4049/jimmunol.1102964 22198953

[23] Yadav M Louvet C Davini D Gardner JM Martinez-Llordella M Bailey-Bucktrout S. Neuropilin-1 distinguishes natural and inducible regulatory T cells among regulatory T cell subsets in vivo. J Exp Med. (2012) 209:1713–22, S1–19. doi: 10.1084/jem.20120822 22966003 PMC3457729

[24] Kim YC Bhairavabhotla R Yoon J Golding A Thornton AM Tran DQ. Oligodeoxynucleotides stabilize helios-expressing foxp3+ Human T regulatory cells during in vitro expansion. Blood. (2012) 119:2810–8. doi: 10.1182/blood-2011-09-377895 PMC3327459 22294730

[25] Koch MA Tucker-Heard G Perdue NR Killebrew JR Urdahl KB Campbell DJ. The transcription factor T-bet controls regulatory T cell homeostasis and function during type 1 inflammation. Nat Immunol. (2009) 10:595–602. doi: 10.1038/ni.1731 19412181 PMC2712126

[26] Kitz A Dominguez-Villar M. Molecular mechanisms underlying th1-like treg generation and function. Cell Mol Life Sci. (2017) 74:4059–75. doi: 10.1007/s00018-017-2569-y PMC7079789 28624966

[27] Krishnamoorthy N Khare A Oriss TB Raundhal M Morse C Yarlagadda M. Early infection with respiratory syncytial virus impairs regulatory T cell function and increases susceptibility to allergic asthma. Nat Med. (2012) 18:1525–30. doi: 10.1038/nm.2896 PMC3641779 22961107

[28] Noval Rivas M Burton OT Wise P Charbonnier LM Georgiev P Oettgen HC. Regulatory T cell reprogramming toward a th2-cell-like lineage impairs oral tolerance and promotes food allergy. Immunity. (2015) 42:512–23. doi: 10.1016/j.immuni.2015.02.004 PMC4366316 25769611

[29] Yao Y Vent-Schmidt J McGeough MD Wong M Hoffman HM Steiner TS. Tr1 cells, but not foxp3+ Regulatory T cells, suppress nlrp3 inflammasome activation via an il-10-dependent mechanism. J Immunol. (2015) 195:488–97. doi: 10.4049/jimmunol.1403225 26056255

[30] Song Y Wang N Chen L Fang L. Tr1 cells as a key regulator for maintaining immune homeostasis in transplantation. Front Immunol. (2021) 12:671579. doi: 10.3389/fimmu.2021.671579 33981317 PMC8109434

[31] Chaturvedi V Collison LW Guy CS Workman CJ Vignali DA. Cutting edge: human regulatory T cells require il-35 to mediate suppression and infectious tolerance. J Immunol. (2011) 186:6661–6. doi: 10.4049/jimmunol.1100315 PMC3110563 21576509

[32] Thornton AM Lu J Korty PE Kim YC Martens C Sun PD. Helios(+) and helios(-) treg subpopulations are phenotypically and functionally distinct and express dissimilar tcr repertoires. Eur J Immunol. (2019) 49:398–412. doi: 10.1002/eji.201847935 30620397 PMC6402968

[33] Wang J Zhao X Wan YY. Intricacies of tgf-beta signaling in treg and th17 cell biology. Cell Mol Immunol. (2023) 20:1002–22. doi: 10.1038/s41423-023-01036-7 PMC10468540 37217798

[34] Venuprasad K Kong YC Farrar MA. Control of th2-mediated inflammation by regulatory T cells. Am J Pathol. (2010) 177:525–31. doi: 10.2353/ajpath.2010.090936 PMC2913351 20566752

[35] Gocher-Demske AM Cui J Szymczak-Workman AL Vignali KM Latini JN Pieklo GP. Ifngamma-induction of T(H)1-like regulatory T cells controls antiviral responses. Nat Immunol. (2023) 24:841–54. doi: 10.1038/s41590-023-01453-w PMC10224582 36928412

[36] Paust HJ Riedel JH Krebs CF Turner JE Brix SR Krohn S. Cxcr3+ Regulatory T cells control th1 responses in crescentic gn. J Am Soc Nephrol. (2016) 27:1933–42. doi: 10.1681/ASN.2015020203 PMC4926966 26534920

[37] Butcher MJ Zhu J. Recent advances in understanding the th1/th2 effector choice. Fac Rev. (2021) 10:30. doi: 10.12703/r/10-30 33817699 PMC8009194

[38] Chapoval S Dasgupta P Dorsey NJ Keegan AD. Regulation of the T helper cell type 2 (Th2)/T regulatory cell (Treg) balance by il-4 and stat6. J Leukoc Biol. (2010) 87:1011–8. doi: 10.1189/jlb.1209772 PMC2872539 20335310

[39] Travis MA Sheppard D. Tgf-beta activation and function in immunity. Annu Rev Immunol. (2014) 32:51–82. doi: 10.1146/annurev-immunol-032713-120257 24313777 PMC4010192

[40] Hata A Chen YG. Tgf-beta signaling from receptors to smads. Cold Spring Harb Perspect Biol. (2016) 8:a022061. doi: 10.1101/cshperspect.a022061 27449815 PMC5008074

[41] Itoh S ten Dijke P. Negative regulation of tgf-beta receptor/smad signal transduction. Curr Opin Cell Biol. (2007) 19:176–84. doi: 10.1016/j.ceb.2007.02.015 17317136

[42] Hanyu A Ishidou Y Ebisawa T Shimanuki T Imamura T Miyazono K. The N domain of smad7 is essential for specific inhibition of transforming growth factor-beta signaling. J Cell Biol. (2001) 155:1017–27. doi: 10.1083/jcb.200106023 PMC2150897 11739411

[43] Galvin KM Donovan MJ Lynch CA Meyer RI Paul RJ Lorenz JN. A role for smad6 in development and homeostasis of the cardiovascular system. Nat Genet. (2000) 24:171–4. doi: 10.1038/72835 10655064

[44] Laudisi F Stolfi C Monteleone I Monteleone G. Tgf-beta1 signaling and smad7 control T-cell responses in health and immune-mediated disorders. Eur J Immunol. (2023) 53:e2350460. doi: 10.1002/eji.202350460 37611637

[45] Lee GR. The balance of th17 versus treg cells in autoimmunity. Int J Mol Sci. (2018) 19:730. doi: 10.3390/ijms19030730 29510522 PMC5877591

[46] Ivanov II Zhou L Littman DR. Transcriptional regulation of th17 cell differentiation. Semin Immunol. (2007) 19:409–17. doi: 10.1016/j.smim.2007.10.011 PMC2696342 18053739

[47] Yang XO Nurieva R Martinez GJ Kang HS Chung Y Pappu BP. Molecular antagonism and plasticity of regulatory and inflammatory T cell programs. Immunity. (2008) 29:44–56. doi: 10.1016/j.immuni.2008.05.007 18585065 PMC2630532

[48] Zhou L Lopes JE Chong MM Ivanov II Min R Victora GD. Tgf-beta-induced foxp3 inhibits T(H)17 cell differentiation by antagonizing rorgammat function. Nature. (2008) 453:236–40. doi: 10.1038/nature06878 PMC2597437 18368049

[49] Tone Y Furuuchi K Kojima Y Tykocinski ML Greene MI Tone M. Smad3 and nfat cooperate to induce foxp3 expression through its enhancer. Nat Immunol. (2008) 9:194–202. doi: 10.1038/ni1549 18157133

[50] Gu AD Wang Y Lin L Zhang SS Wan YY. Requirements of transcription factor smad-dependent and -independent tgf-beta signaling to control discrete T-cell functions. Proc Natl Acad Sci U.S.A. (2012) 109:905–10. doi: 10.1073/pnas.1108352109 PMC3271895 22219364

[51] Zhang W Zhang D Shen M Liu Y Tian Y Thomson AW. Combined administration of a mutant tgf-beta1/fc and rapamycin promotes induction of regulatory T cells and islet allograft tolerance. J Immunol. (2010) 185:4750–9. doi: 10.4049/jimmunol.1000769 PMC3766970 20844194

[52] Kay JE Kromwel L Doe SE Denyer M. Inhibition of T and B lymphocyte proliferation by rapamycin. Immunology. (1991) 72:544–9. PMC1384375 1709916

[53] Zhang F Cheng T Zhang SX. Mechanistic target of rapamycin (Mtor): A potential new therapeutic target for rheumatoid arthritis. Arthritis Res Ther. (2023) 25:187. doi: 10.1186/s13075-023-03181-w 37784141 PMC10544394

[54] Li Z Nie L Chen L Sun Y Li G. Rapamycin relieves inflammation of experimental autoimmune encephalomyelitis by altering the balance of treg/th17 in a mouse model. Neurosci Lett. (2019) 705:39–45. doi: 10.1016/j.neulet.2019.04.035 31004709

[55] Chen SY Mamai O Akhurst RJ. Tgfbeta: signaling blockade for cancer immunotherapy. Annu Rev Cancer Biol. (2022) 6:123–46. doi: 10.1146/annurev-cancerbio-070620-103554 PMC9645596 36382146

[56] Luo Q Hu Z Zhao H Fan Y Tu X Wang Y. The role of tgf-beta in the tumor microenvironment of pancreatic cancer. Genes Dis. (2023) 10:1513–24. doi: 10.1016/j.gendis.2022.10.019 PMC10311106 37397548

[57] Tao B Yi C Ma Y Li Y Zhang B Geng Y. A novel tgf-beta-related signature for predicting prognosis, tumor microenvironment, and therapeutic response in colorectal cancer. Biochem Genet. (2023). doi: 10.1007/s10528-023-10591-7 [Epub ahead of print] 38062276

[58] Bachmann MF Oxenius A. Interleukin 2: from immunostimulation to immunoregulation and back again. EMBO Rep. (2007) 8:1142–8. doi: 10.1038/sj.embor.7401099 PMC2267244 18059313

[59] Abbas AK Trotta E D Marson A Bluestone JA. Revisiting il-2: biology and therapeutic prospects. Sci Immunol. (2018) 3:eaat1482. doi: 10.1126/sciimmunol.aat1482 29980618

[60] Smith GA Uchida K Weiss A Taunton J. Essential biphasic role for jak3 catalytic activity in il-2 receptor signaling. Nat Chem Biol. (2016) 12:373–9. doi: 10.1038/nchembio.2056 PMC4837022 27018889

[61] Ross SH Cantrell DA. Signaling and function of interleukin-2 in T lymphocytes. Annu Rev Immunol. (2018) 36:411–33. doi: 10.1146/annurev-immunol-042617-053352 PMC6472684 29677473

[62] Liao W Lin JX Leonard WJ. Interleukin-2 at the crossroads of effector responses, tolerance, and immunotherapy. Immunity. (2013) 38:13–25. doi: 10.1016/j.immuni.2013.01.004 23352221 PMC3610532

[63] Feng Y Arvey A Chinen T van der Veeken J Gasteiger G Rudensky AY. Control of the inheritance of regulatory T cell identity by a cis element in the foxp3 locus. Cell. (2014) 158:749–63. doi: 10.1016/j.cell.2014.07.031 PMC4151558 25126783

[64] Yu A Snowhite I Vendrame F Rosenzwajg M Klatzmann D Pugliese A. Selective il-2 responsiveness of regulatory T cells through multiple intrinsic mechanisms supports the use of low-dose il-2 therapy in type 1 diabetes. Diabetes. (2015) 64:2172–83. doi: 10.2337/db14-1322 25576057

[65] Sezin T Selvakumar B Scheffold A. The role of a disintegrin and metalloproteinase (Adam)-10 in T helper cell biology. Biochim Biophys Acta Mol Cell Res. (2022) 1869:119192. doi: 10.1016/j.bbamcr.2021.119192 34982961

[66] Tomala J Weberova P Tomalova B Jiraskova Zakostelska Z Sivak L Kovarova J. Il-2/jes6–1 mab complexes dramatically increase sensitivity to lps through ifn-gamma production by cd25(+)Foxp3(-) T cells. Elife. (2021) 10:e62432. doi: 10.7554/eLife.62432 34932467 PMC8691839

[67] Betts BC Pidala J Kim J Mishra A Nishihori T Perez L. Il-2 promotes early treg reconstitution after allogeneic hematopoietic cell transplantation. Haematologica. (2017) 102:948–57. doi: 10.3324/haematol.2016.153072 PMC5477614 28104702

[68] Saadoun D Rosenzwajg M Joly F Six A Carrat F Thibault V. Regulatory T-cell responses to low-dose interleukin-2 in hcv-induced vasculitis. N Engl J Med. (2011) 365:2067–77. doi: 10.1056/NEJMoa1105143 22129253

[69] Hartemann A Bensimon G Payan CA Jacqueminet S Bourron O Nicolas N. Low-dose interleukin 2 in patients with type 1 diabetes: A phase 1/2 randomised, double-blind, placebo-controlled trial. Lancet Diabetes Endocrinol. (2013) 1:295–305. doi: 10.1016/S2213-8587(13)70113-X 24622415

[70] Miao M Hao Z Guo Y Zhang X Zhang S Luo J. Short-term and low-dose il-2 therapy restores the th17/treg balance in the peripheral blood of patients with primary Sjogren’s syndrome. Ann Rheum Dis. (2018) 77:1838–40. doi: 10.1136/annrheumdis-2018-213036 29936436

[71] Zhang SX Wang J Sun HH Zhang JQ Liu GY Luo J. Circulating regulatory T cells were absolutely decreased in dermatomyositis/polymyositis patients and restored by low-dose il-2. Ann Rheum Dis. (2021) 80:e130. doi: 10.1136/annrheumdis-2019-216246 31611221

[72] Rosenzwajg M Lorenzon R Cacoub P Pham HP Pitoiset F El Soufi K. Immunological and clinical effects of low-dose interleukin-2 across 11 autoimmune diseases in a single, open clinical trial. Ann Rheum Dis. (2019) 78:209–17. doi: 10.1136/annrheumdis-2018-214229 30472651

[73] Moore KW de Waal Malefyt R Coffman RL O’Garra A. Interleukin-10 and the interleukin-10 receptor. Annu Rev Immunol. (2001) 19:683–765. doi: 10.1146/annurev.immunol.19.1.683 11244051

[74] Riley JK Takeda K Akira S Schreiber RD. Interleukin-10 receptor signaling through the jak-stat pathway. Requirement for two distinct receptor-derived signals for anti-inflammatory action. J Biol Chem. (1999) 274:16513–21. doi: 10.1074/jbc.274.23.16513 10347215

[75] Wingelhofer B Neubauer HA Valent P Han X Constantinescu SN Gunning PT. Implications of stat3 and stat5 signaling on gene regulation and chromatin remodeling in hematopoietic cancer. Leukemia. (2018) 32:1713–26. doi: 10.1038/s41375-018-0117-x PMC6087715 29728695

[76] Hammer M Mages J Dietrich H Schmitz F Striebel F Murray PJ. Control of dual-specificity phosphatase-1 expression in activated macrophages by il-10. Eur J Immunol. (2005) 35:2991–3001. doi: 10.1002/eji.200526192 16184516

[77] Powell MJ Thompson SA Tone Y Waldmann H Tone M. Posttranscriptional regulation of il-10 gene expression through sequences in the 3’-untranslated region. J Immunol. (2000) 165:292–6. doi: 10.4049/jimmunol.165.1.292 10861064

[78] Schulte LN Eulalio A Mollenkopf HJ Reinhardt R Vogel J. Analysis of the host microrna response to salmonella uncovers the control of major cytokines by the let-7 family. EMBO J. (2011) 30:1977–89. doi: 10.1038/emboj.2011.94 PMC3098495 21468030

[79] Sharma A Kumar M Aich J Hariharan M Brahmachari SK Agrawal A. Posttranscriptional regulation of interleukin-10 expression by hsa-mir-106a. Proc Natl Acad Sci U.S.A. (2009) 106:5761–6. doi: 10.1073/pnas.0808743106 PMC2659714 19307576

[80] Cheng H Wang L Yang B Li D Wang X Liu X. Cutting edge: inhibition of glycogen synthase kinase 3 activity induces the generation and enhanced suppressive function of human il-10(+) foxp3(+)-induced regulatory T cells. J Immunol. (2020) 205:1497–502. doi: 10.4049/jimmunol.2000136 PMC7477744 32817370

[81] Chihara N Madi A Karwacz K Awasthi A Kuchroo VK. Differentiation and characterization of tr1 cells. Curr Protoc Immunol. (2016) 113:3 27 1–3 10. doi: 10.1002/0471142735.im0327s113 PMC5933847 27038462

[82] Apetoh L Quintana FJ Pot C Joller N Xiao S Kumar D. The aryl hydrocarbon receptor interacts with C-maf to promote the differentiation of type 1 regulatory T cells induced by il-27. Nat Immunol. (2010) 11:854–61. doi: 10.1038/ni.1912 PMC2940320 20676095

[83] Brockmann L Soukou S Steglich B Czarnewski P Zhao L Wende S. Molecular and functional heterogeneity of il-10-producing cd4(+) T cells. Nat Commun. (2018) 9:5457. doi: 10.1038/s41467-018-07581-4 30575716 PMC6303294

[84] Ranjbar M Solgi G Mohammadnia M Nikbin B Pourmand G Ansaripour B. Regulatory T-cell subset analysis and profile of interleukin (Il)-10, il-17 and interferon-gamma cytokine-producing cells in kidney allograft recipients with donor cells infusion. Clin Exp Nephrol. (2012) 16:636–46. doi: 10.1007/s10157-012-0591-9 22314659

[85] Singh A Ramachandran S Graham ML Daneshmandi S Heller D Suarez-Pinzon WL. Long-term tolerance of islet allografts in nonhuman primates induced by apoptotic donor leukocytes. Nat Commun. (2019) 10:3495. doi: 10.1038/s41467-019-11338-y 31375697 PMC6677762

[86] Riley JS McClain LE Stratigis JD Coons BE Ahn NJ Li H. Regulatory T cells promote alloengraftment in a model of late-gestation in utero hematopoietic cell transplantation. Blood Adv. (2020) 4:1102–14. doi: 10.1182/bloodadvances.2019001208 PMC7094012 32203584

[87] Fitzgerald DC Zhang GX El-Behi M Fonseca-Kelly Z Li H Yu S. Suppression of autoimmune inflammation of the central nervous system by interleukin 10 secreted by interleukin 27-stimulated T cells. Nat Immunol. (2007) 8:1372–9. doi: 10.1038/ni1540 17994023

[88] Chong WP van Panhuys N Chen J Silver PB Jittayasothorn Y Mattapallil MJ. Nk-dc crosstalk controls the autopathogenic th17 response through an innate ifn-gamma-il-27 axis. J Exp Med. (2015) 212:1739–52. doi: 10.1084/jem.20141678 PMC4577839 26347474

[89] Saraiva M Vieira P O’Garra A. Biology and therapeutic potential of interleukin-10. J Exp Med. (2020) 217:e20190418. doi: 10.1084/jem.20190418 31611251 PMC7037253

[90] Armstrong L Jordan N Millar A. Interleukin 10 (Il-10) regulation of tumour necrosis factor alpha (Tnf-alpha) from human alveolar macrophages and peripheral blood monocytes. Thorax. (1996) 51:143–9. doi: 10.1136/thx.51.2.143 PMC473022 8711645

[91] Saraiva M O’Garra A. The regulation of il-10 production by immune cells. Nat Rev Immunol. (2010) 10:170–81. doi: 10.1038/nri2711 20154735

[92] Rivas JR Liu Y Alhakeem SS Eckenrode JM Marti F Collard JP. Interleukin-10 suppression enhances T-cell antitumor immunity and responses to checkpoint blockade in chronic lymphocytic leukemia. Leukemia. (2021) 35:3188–200. doi: 10.1038/s41375-021-01217-1 PMC8446094 33731852

[93] Beppu LY Mooli RGR Qu X Marrero GJ Finley CA Fooks AN. Tregs facilitate obesity and insulin resistance via a blimp-1/il-10 axis. JCI Insight. (2021) 6:e140644. doi: 10.1172/jci.insight.140644 33351782 PMC7934851

[94] Mosmann TR Moore KW. The role of il-10 in crossregulation of th1 and th2 responses. Immunol Today. (1991) 12:A49–53. doi: 10.1016/S0167-5699(05)80015-5 1648926

[95] Allavena P Piemonti L Longoni D Bernasconi S Stoppacciaro A Ruco L. Il-10 prevents the differentiation of monocytes to dendritic cells but promotes their maturation to macrophages. Eur J Immunol. (1998) 28:359–69. doi: 10.1002/(SICI)1521-4141(199801)28:01<359::AID-IMMU359>3.0.CO;2-4 9485215

[96] Koch F Stanzl U Jennewein P Janke K Heufler C Kampgen E. High level il-12 production by murine dendritic cells: upregulation via mhc class ii and cd40 molecules and downregulation by il-4 and il-10. J Exp Med. (1996) 184:741–6. doi: 10.1084/jem.184.2.741 PMC2192732 8760828

[97] Mittal SK Roche PA. Suppression of antigen presentation by il-10. Curr Opin Immunol. (2015) 34:22–7. doi: 10.1016/j.coi.2014.12.009 PMC4444374 25597442

[98] Carter NA Vasconcellos R Rosser EC Tulone C Munoz-Suano A Kamanaka M. Mice lacking endogenous il-10-producing regulatory B cells develop exacerbated disease and present with an increased frequency of th1/th17 but a decrease in regulatory T cells. J Immunol. (2011) 186:5569–79. doi: 10.4049/jimmunol.1100284 21464089

[99] Groux H Cottrez F Rouleau M Mauze S Antonenko S Hurst S. A transgenic model to analyze the immunoregulatory role of il-10 secreted by antigen-presenting cells. J Immunol. (1999) 162:1723–9. 9973435

[100] Fujii S Shimizu K Shimizu T Lotze MT. Interleukin-10 promotes the maintenance of antitumor cd8(+) T-cell effector function in situ. Blood. (2001) 98:2143–51. doi: 10.1182/blood.v98.7.2143 11568001

[101] Emmerich J Mumm JB Chan IH LaFace D Truong H McClanahan T. Il-10 directly activates and expands tumor-resident cd8(+) T cells without *de novo* infiltration from secondary lymphoid organs. Cancer Res. (2012) 72:3570–81. doi: 10.1158/0008-5472.CAN-12-0721 22581824

[102] Collison LW Delgoffe GM Guy CS Vignali KM Chaturvedi V Fairweather D. The composition and signaling of the il-35 receptor are unconventional. Nat Immunol. (2012) 13:290–9. doi: 10.1038/ni.2227 PMC3529151 22306691

[103] Zhang J Zhang Y Wang Q Li C Deng H Si C. Interleukin-35 in immune-related diseases: protection or destruction. Immunology. (2019) 157:13–20. doi: 10.1111/imm.13044 30681737 PMC6459776

[104] Dong Y Li X Yu Y Lv F Chen Y. Jak/stat signaling is involved in il-35-induced inhibition of hepatitis B virus antigen-specific cytotoxic T cell exhaustion in chronic hepatitis B. Life Sci. (2020) 252:117663. doi: 10.1016/j.lfs.2020.117663 32302624

[105] Zysk W Glen J Trzeciak M. Current insight into the role of il-35 and its potential involvement in the pathogenesis and therapy of atopic dermatitis. Int J Mol Sci. (2022) 23:15709. doi: 10.3390/ijms232415709 36555351 PMC9779445

[106] Collison LW Workman CJ Kuo TT Boyd K Wang Y Vignali KM. The inhibitory cytokine il-35 contributes to regulatory T-cell function. Nature. (2007) 450:566–9. doi: 10.1038/nature06306 18033300

[107] Zeng JC Zhang Z Li TY Liang YF Wang HM Bao JJ. Assessing the role of il-35 in colorectal cancer progression and prognosis. Int J Clin Exp Pathol. (2013) 6:1806–16. PMC3759487 24040445

[108] Wang Z Liu JQ Liu Z Shen R Zhang G Xu J. Tumor-derived il-35 promotes tumor growth by enhancing myeloid cell accumulation and angiogenesis. J Immunol. (2013) 190:2415–23. doi: 10.4049/jimmunol.1202535 PMC3578001 23345334

[109] Turnis ME Sawant DV Szymczak-Workman AL Andrews LP Delgoffe GM Yano H. Interleukin-35 limits anti-tumor immunity. Immunity. (2016) 44:316–29. doi: 10.1016/j.immuni.2016.01.013 PMC4758699 26872697

[110] Tanaka T Narazaki M Kishimoto T. Il-6 in inflammation, immunity, and disease. Cold Spring Harb Perspect Biol. (2014) 6:a016295. doi: 10.1101/cshperspect.a016295 25190079 PMC4176007

[111] Fujimoto M Nakano M Terabe F Kawahata H Ohkawara T Han Y. The influence of excessive il-6 production in vivo on the development and function of foxp3+ Regulatory T cells. J Immunol. (2011) 186:32–40. doi: 10.4049/jimmunol.0903314 21106853

[112] Koenecke C Lee CW Thamm K Fohse L Schafferus M Mittrucker HW. Ifn-gamma production by allogeneic foxp3+ Regulatory T cells is essential for preventing experimental graft-versus-host disease. J Immunol. (2012) 189:2890–6. doi: 10.4049/jimmunol.1200413 22869903

[113] Bettelli E Carrier Y Gao W Korn T Strom TB Oukka M. Reciprocal developmental pathways for the generation of pathogenic effector th17 and regulatory T cells. Nature. (2006) 441:235–8. doi: 10.1038/nature04753 16648838

[114] Sousa RO Cariaco Y Almeida MPO Nascimento LAC Coutinho LB Ferreira-Junior AA. The imbalance of tnf and il-6 levels and foxp3 expression at the maternal-fetal interface is involved in adverse pregnancy outcomes in a susceptible murine model of congenital toxoplasmosis. Cytokine. (2021) 143:155517. doi: 10.1016/j.cyto.2021.155517 33814270

[115] Xu L Kitani A Fuss I Strober W. Cutting edge: regulatory T cells induce cd4+Cd25-foxp3- T cells or are self-induced to become th17 cells in the absence of exogenous tgf-beta. J Immunol. (2007) 178:6725–9. doi: 10.4049/jimmunol.178.11.6725 17513718

[116] Zhang T Han X Zhong Y Kam HT Qiao D Chen Z. Regulatory T cell intravitreal delivery using hyaluronan methylcellulose hydrogel improves therapeutic efficacy in experimental autoimmune uveitis. Biomater Adv. (2023) 151:213496. doi: 10.1016/j.bioadv.2023.213496 37290283

[117] Kimura A Kishimoto T. Il-6: regulator of treg/th17 balance. Eur J Immunol. (2010) 40:1830–5. doi: 10.1002/eji.201040391 20583029

[118] Bettelli E Korn T Oukka M Kuchroo VK. Induction and effector functions of T(H)17 cells. Nature. (2008) 453:1051–7. doi: 10.1038/nature07036 PMC6280661 18563156

[119] Ogura H Murakami M Okuyama Y Tsuruoka M Kitabayashi C Kanamoto M. Interleukin-17 promotes autoimmunity by triggering a positive-feedback loop via interleukin-6 induction. Immunity. (2008) 29:628–36. doi: 10.1016/j.immuni.2008.07.018 18848474

[120] Yang XO Panopoulos AD Nurieva R Chang SH Wang D Watowich SS. Stat3 regulates cytokine-mediated generation of inflammatory helper T cells. J Biol Chem. (2007) 282:9358–63. doi: 10.1074/jbc.C600321200 17277312

[121] Wang Y van Boxel-Dezaire AH Cheon H Yang J Stark GR. Stat3 activation in response to il-6 is prolonged by the binding of il-6 receptor to egf receptor. Proc Natl Acad Sci U.S.A. (2013) 110:16975–80. doi: 10.1073/pnas.1315862110 PMC3801081 24082147

[122] Zhang W Liu X Zhu Y Liu X Gu Y Dai X. Transcriptional and posttranslational regulation of th17/treg balance in health and disease. Eur J Immunol. (2021) 51:2137–50. doi: 10.1002/eji.202048794 34322865

[123] Iliopoulos D Hirsch HA Struhl K. An epigenetic switch involving nf-kappab, lin28, let-7 microrna, and il6 links inflammation to cell transformation. Cell. (2009) 139:693–706. doi: 10.1016/j.cell.2009.10.014 19878981 PMC2783826

[124] Guichelaar T Emmelot ME Rozemuller H Martini B Groen RW Storm G. Human regulatory T cells do not suppress the antitumor immunity in the bone marrow: A role for bone marrow stromal cells in neutralizing regulatory T cells. Clin Cancer Res. (2013) 19:1467–75. doi: 10.1158/1078-0432.CCR-12-2177 23382115

[125] Lonial S Durie B Palumbo A San-Miguel J. Monoclonal antibodies in the treatment of multiple myeloma: current status and future perspectives. Leukemia. (2016) 30:526–35. doi: 10.1038/leu.2015.223 PMC4777772 26265184

[126] Hunsucker SA Magarotto V Kuhn DJ Kornblau SM Wang M Weber DM. Blockade of interleukin-6 signalling with siltuximab enhances melphalan cytotoxicity in preclinical models of multiple myeloma. Br J Haematol. (2011) 152:579–92. doi: 10.1111/j.1365-2141.2010.08533.x PMC3402914 21241278

[127] Xiao S Jin H Korn T Liu SM Oukka M Lim B. Retinoic acid increases foxp3+ Regulatory T cells and inhibits development of th17 cells by enhancing tgf-beta-driven smad3 signaling and inhibiting il-6 and il-23 receptor expression. J Immunol. (2008) 181:2277–84. doi: 10.4049/jimmunol.181.4.2277 PMC2722959 18684916

[128] Valencia X Stephens G Goldbach-Mansky R Wilson M Shevach EM Lipsky PE. Tnf downmodulates the function of human cd4+Cd25hi T-regulatory cells. Blood. (2006) 108:253–61. doi: 10.1182/blood-2005-11-4567 PMC1895836 16537805

[129] Zhang Q Cui F Fang L Hong J Zheng B Zhang JZ. Tnf-alpha impairs differentiation and function of tgf-beta-induced treg cells in autoimmune diseases through akt and smad3 signaling pathway. J Mol Cell Biol. (2013) 5:85–98. doi: 10.1093/jmcb/mjs063 23243069

[130] Chen X Baumel M Mannel DN Howard OM Oppenheim JJ. Interaction of tnf with tnf receptor type 2 promotes expansion and function of mouse cd4+Cd25+ T regulatory cells. J Immunol. (2007) 179:154–61. doi: 10.4049/jimmunol.179.1.154 17579033

[131] Hamano R Huang J Yoshimura T Oppenheim JJ Chen X. Tnf optimally activatives regulatory T cells by inducing tnf receptor superfamily members tnfr2, 4–1bb and ox40. Eur J Immunol. (2011) 41:2010–20. doi: 10.1002/eji.201041205 PMC3783213 21491419

[132] Chen X Wu X Zhou Q Howard O Netea MG Oppenheim JJ. Tnfr2 is critical for the stabilization of the cd4+ Foxp3+ Regulatory T cell phenotype in the inflammatory environment. J Immunol. (2013) 190:1076–84. doi: 10.4049/jimmunol.1202659 PMC3552130 23277487

[133] Mahmud SA Manlove LS Schmitz HM Xing Y Wang Y Owen DL. Costimulation via the tumor-necrosis factor receptor superfamily couples tcr signal strength to the thymic differentiation of regulatory T cells. Nat Immunol. (2014) 15:473–81. doi: 10.1038/ni.2849 PMC4000541 24633226

[134] Evans HG Roostalu U Walter GJ Gullick NJ Frederiksen KS Roberts CA. Tnf-alpha blockade induces il-10 expression in human cd4+ T cells. Nat Commun. (2014) 5:3199. doi: 10.1038/ncomms4199 24492460 PMC3918582

[135] Holtta V Sipponen T Westerholm-Ormio M Salo HM Kolho KL Farkkila M. In crohn’s disease, anti-tnf-alpha treatment changes the balance between mucosal il-17, foxp3, and cd4 cells. ISRN Gastroenterol. (2012) 2012:505432. doi: 10.5402/2012/505432 22778976 PMC3384926

[136] Ternant D Pfister M Le Tilly O Mulleman D Picon L Willot S. Infliximab treatment does not lead to full tnf-alpha inhibition: A target-mediated drug disposition model. Clin Pharmacokinet. (2022) 61:143–54. doi: 10.1007/s40262-021-01057-3 34351609

[137] Nie H Zheng Y Li R Guo TB He D Fang L. Phosphorylation of foxp3 controls regulatory T cell function and is inhibited by tnf-alpha in rheumatoid arthritis. Nat Med. (2013) 19:322–8. doi: 10.1038/nm.3085 23396208

[138] Dwivedi M Tiwari S Kemp EH Begum R. Implications of regulatory T cells in anti-cancer immunity: from pathogenesis to therapeutics. Heliyon. (2022) 8:e10450. doi: 10.1016/j.heliyon.2022.e10450 36082331 PMC9445387

[139] Iglesias-Escudero M Arias-Gonzalez N Martinez-Caceres E. Regulatory cells and the effect of cancer immunotherapy. Mol Cancer. (2023) 22:26. doi: 10.1186/s12943-023-01714-0 36739406 PMC9898962

[140] Guo Y Xie F Liu X Ke S Chen J Zhao Y. Blockade of tnf-alpha/tnfr2 signalling suppresses colorectal cancer and enhances the efficacy of anti-pd1 immunotherapy by decreasing ccr8+ T regulatory cells. J Mol Cell Biol. (2023). doi: 10.1093/jmcb/mjad067 [Epub ahead of print] PMC11587560 37935468

[141] Qu Y Wang X Bai S Niu L Zhao G Yao Y. The effects of tnf-alpha/tnfr2 in regulatory T cells on the microenvironment and progression of gastric cancer. Int J Cancer. (2022) 150:1373–91. doi: 10.1002/ijc.33873 PMC9298834 34766338

[142] Greenwald RJ Freeman GJ Sharpe AH. The B7 family revisited. Annu Rev Immunol. (2005) 23:515–48. doi: 10.1146/annurev.immunol.23.021704.115611 15771580

[143] Zang X Allison JP. The B7 family and cancer therapy: costimulation and coinhibition. Clin Cancer Res. (2007) 13:5271–9. doi: 10.1158/1078-0432.CCR-07-1030 17875755

[144] Wang Y Zhang H Liu C Wang Z Wu W Zhang N. Immune checkpoint modulators in cancer immunotherapy: recent advances and emerging concepts. J Hematol Oncol. (2022) 15:111. doi: 10.1186/s13045-022-01325-0 35978433 PMC9386972

[145] Larkin J Chiarion-Sileni V Gonzalez R Grob JJ Rutkowski P Lao CD. Five-year survival with combined nivolumab and ipilimumab in advanced melanoma. N Engl J Med. (2019) 381:1535–46. doi: 10.1056/NEJMoa1910836 31562797

[146] Babamohamadi M Mohammadi N Faryadi E Haddadi M Merati A Ghobadinezhad F. Anti-ctla-4 nanobody as a promising approach in cancer immunotherapy. Cell Death Dis. (2024) 15:17. doi: 10.1038/s41419-023-06391-x 38191571 PMC10774412

[147] Qureshi OS Zheng Y Nakamura K Attridge K Manzotti C Schmidt EM. Trans-endocytosis of cd80 and cd86: A molecular basis for the cell-extrinsic function of ctla-4. Science. (2011) 332:600–3. doi: 10.1126/science.1202947 PMC3198051 21474713

[148] Waldman AD Fritz JM Lenardo MJ. A guide to cancer immunotherapy: from T cell basic science to clinical practice. Nat Rev Immunol. (2020) 20:651–68. doi: 10.1038/s41577-020-0306-5 PMC7238960 32433532

[149] Paterson AM Lovitch SB Sage PT Juneja VR Lee Y Trombley JD. Deletion of ctla-4 on regulatory T cells during adulthood leads to resistance to autoimmunity. J Exp Med. (2015) 212:1603–21. doi: 10.1084/jem.20141030 PMC4577848 26371185

[150] Jain N Nguyen H Chambers C Kang J. Dual function of ctla-4 in regulatory T cells and conventional T cells to prevent multiorgan autoimmunity. Proc Natl Acad Sci U.S.A. (2010) 107:1524–8. doi: 10.1073/pnas.0910341107 PMC2824392 20080649

[151] Hossen MM Ma Y Yin Z Xia Y Du J Huang JY. Current understanding of ctla-4: from mechanism to autoimmune diseases. Front Immunol. (2023) 14:1198365. doi: 10.3389/fimmu.2023.1198365 37497212 PMC10367421

[152] Shao L Gao Y Shao X Ou Q Zhang S Liu Q. Ctla-4 blockade reverses the foxp3+ T-regulatory-cell suppression of anti-tuberculosis T-cell effector responses. bioRxiV [Preprint] (2020). doi: 10.1101/2020.05.11.089946

[153] Sun HL Du XF Tang YX Li GQ Yang SY Wang LH. Impact of immune checkpoint molecules on foxp3(+) treg cells and related cytokines in patients with acute and chronic brucellosis. BMC Infect Dis. (2021) 21:1025. doi: 10.1186/s12879-021-06730-3 34592958 PMC8482665

[154] Dhunputh C Ducassou S Fernandes H Picard C Rieux-Laucat F Viallard JF. Abatacept is useful in autoimmune cytopenia with immunopathologic manifestations caused by ctla-4 defects. Blood. (2022) 139:300–4. doi: 10.1182/blood.2021013496 34714911

[155] Krummey SM Cheeseman JA Conger JA Jang PS Mehta AK Kirk AD. High ctla-4 expression on th17 cells results in increased sensitivity to ctla-4 coinhibition and resistance to belatacept. Am J Transplant. (2014) 14:607–14. doi: 10.1111/ajt.12600 PMC4124942 24730049

[156] Sobhani N Tardiel-Cyril DR Davtyan A Generali D Roudi R Li Y. Ctla-4 in regulatory T cells for cancer immunotherapy. Cancers (Basel). (2021) 13:1440. doi: 10.3390/cancers13061440 33809974 PMC8005092

[157] Stumpf M Zhou X Bluestone JA. The B7-independent isoform of ctla-4 functions to regulate autoimmune diabetes. J Immunol. (2013) 190:961–9. doi: 10.4049/jimmunol.1201362 PMC3568535 23293354

[158] Vasu C Gorla SR Prabhakar BS Holterman MJ. Targeted engagement of ctla-4 prevents autoimmune thyroiditis. Int Immunol. (2003) 15:641–54. doi: 10.1093/intimm/dxg061 12697664

[159] Fan Q Zhang J Cui Y Wang C Xie Y Wang Q. The synergic effects of ctla-4/foxp3-related genotypes and chromosomal aberrations on the risk of recurrent spontaneous abortion among a Chinese han population. J Hum Genet. (2018) 63:579–87. doi: 10.1038/s10038-018-0414-2 PMC5915418 29476189

[160] Masterman T Ligers A Zhang Z Hellgren D Salter H Anvret M. Ctla4 dimorphisms and the multiple sclerosis phenotype. J Neuroimmunol. (2002) 131:208–12. doi: 10.1016/s0165-5728(02)00274-6 12458054

[161] Parry RV Chemnitz JM Frauwirth KA Lanfranco AR Braunstein I Kobayashi SV. Ctla-4 and pd-1 receptors inhibit T-cell activation by distinct mechanisms. Mol Cell Biol. (2005) 25:9543–53. doi: 10.1128/MCB.25.21.9543-9553.2005 PMC1265804 16227604

[162] Farhangnia P Ghomi SM Akbarpour M Delbandi AA. Bispecific antibodies targeting ctla-4: game-changer troopers in cancer immunotherapy. Front Immunol. (2023) 14:1155778. doi: 10.3389/fimmu.2023.1155778 37441075 PMC10333476

[163] Chen RY Zhu Y Shen YY Xu QY Tang HY Cui NX. The role of pd-1 signaling in health and immune-related diseases. Front Immunol. (2023) 14:1163633. doi: 10.3389/fimmu.2023.1163633 37261359 PMC10228652

[164] Gianchecchi E Fierabracci A. Inhibitory receptors and pathways of lymphocytes: the role of pd-1 in treg development and their involvement in autoimmunity onset and cancer progression. Front Immunol. (2018) 9:2374. doi: 10.3389/fimmu.2018.02374 30386337 PMC6199356

[165] Yamaguchi H Hsu JM Yang WH Hung MC. Mechanisms regulating pd-L1 expression in cancers and associated opportunities for novel small-molecule therapeutics. Nat Rev Clin Oncol. (2022) 19:287–305. doi: 10.1038/s41571-022-00601-9 35132224

[166] Pauken KE Torchia JA Chaudhri A Sharpe AH Freeman GJ. Emerging concepts in pd-1 checkpoint biology. Semin Immunol. (2021) 52:101480. doi: 10.1016/j.smim.2021.101480 34006473 PMC8545711

[167] Lowther DE Goods BA Lucca LE Lerner BA Raddassi K van Dijk D. Pd-1 marks dysfunctional regulatory T cells in Malignant gliomas. JCI Insight. (2016) 1:e85935. doi: 10.1172/jci.insight.85935 27182555 PMC4864991

[168] Tan CL Kuchroo JR Sage PT Liang D Francisco LM Buck J. Pd-1 restraint of regulatory T cell suppressive activity is critical for immune tolerance. J Exp Med. (2021) 218:e20182232. doi: 10.1084/jem.20182232 33045061 PMC7543091

[169] Kumagai S Togashi Y Kamada T Sugiyama E Nishinakamura H Takeuchi Y. The pd-1 expression balance between effector and regulatory T cells predicts the clinical efficacy of pd-1 blockade therapies. Nat Immunol. (2020) 21:1346–58. doi: 10.1038/s41590-020-0769-3 32868929

[170] Deng B Yang B Chen J Wang S Zhang W Guo Y. Gallic acid induces T-helper-1-like T(Reg) cells and strengthens immune checkpoint blockade efficacy. J Immunother Cancer. (2022) 10:e004037. doi: 10.1136/jitc-2021-004037 35817479 PMC9274539

[171] Sage PT Francisco LM Carman CV Sharpe AH. The receptor pd-1 controls follicular regulatory T cells in the lymph nodes and blood. Nat Immunol. (2013) 14:152–61. doi: 10.1038/ni.2496 PMC3788614 23242415

[172] Chuckran CA Liu C Bruno TC Workman CJ Vignali DA. Neuropilin-1: A checkpoint target with unique implications for cancer immunology and immunotherapy. J Immunother Cancer. (2020) 8:e000967. doi: 10.1136/jitc-2020-000967 32675311 PMC7368550

[173] Graziani G Lacal PM. Neuropilin-1 as therapeutic target for Malignant melanoma. Front Oncol. (2015) 5:125. doi: 10.3389/fonc.2015.00125 26090340 PMC4453476

[174] Yang X Xu T Song X Wu Y. Overexpression of nrp1 is associated with poor prognosis via accelerating immunosuppression in head and neck squamous cell carcinoma. Int J Gen Med. (2023) 16:2819–29. doi: 10.2147/IJGM.S409336 PMC10329464 37426519

[175] Liu Z McMichael EL Shayan G Li J Chen K Srivastava R. Novel effector phenotype of tim-3(+) regulatory T cells leads to enhanced suppressive function in head and neck cancer patients. Clin Cancer Res. (2018) 24:4529–38. doi: 10.1158/1078-0432.CCR-17-1350 PMC6139056 29712685

[176] Khunger M Hernandez AV Pasupuleti V Rakshit S Pennell NA Stevenson J. Programmed cell death 1 (Pd-1) ligand (Pd-L1) expression in solid tumors as a predictive biomarker of benefit from pd-1/pd-L1 axis inhibitors: A systematic review and meta-analysis. JCO Precis Oncol. (2017) 1:1–15. doi: 10.1200/PO.16.00030 35172490

[177] Zhang B Liu Y Zhou S Jiang H Zhu K Wang R. Predictive effect of pd-L1 expression for immune checkpoint inhibitor (Pd-1/pd-L1 inhibitors) treatment for non-small cell lung cancer: A meta-analysis. Int Immunopharmacol. (2020) 80:106214. doi: 10.1016/j.intimp.2020.106214 31982822

[178] Ghobadinezhad F Ebrahimi N Mozaffari F Moradi N Beiranvand S Pournazari M. The emerging role of regulatory cell-based therapy in autoimmune disease. Front Immunol. (2022) 13:1075813. doi: 10.3389/fimmu.2022.1075813 36591309 PMC9795194

[179] Spence A Klementowicz JE Bluestone JA Tang Q. Targeting treg signaling for the treatment of autoimmune diseases. Curr Opin Immunol. (2015) 37:11–20. doi: 10.1016/j.coi.2015.09.002 26432763 PMC4679451

[180] Park TY Jeon J Lee N Kim J Song B Kim JH. Co-transplantation of autologous T(Reg) cells in a cell therapy for Parkinson’s disease. Nature. (2023) 619:606–15. doi: 10.1038/s41586-023-06300-4 PMC12012854 37438521

[181] Daenthanasanmak A Iamsawat S Chakraborty P Nguyen HD Bastian D Liu C. Targeting sirt-1 controls gvhd by inhibiting T-cell allo-response and promoting treg stability in mice. Blood. (2019) 133:266–79. doi: 10.1182/blood-2018-07-863233 PMC6337874 30514750

[182] Hosseinalizadeh H Rabiee F Eghbalifard N Rajabi H Klionsky DJ Rezaee A. Regulating the regulatory T cells as cell therapies in autoimmunity and cancer. Front Med (Lausanne). (2023) 10:1244298. doi: 10.3389/fmed.2023.1244298 37828948 PMC10565010

[183] Andre S Tough DF Lacroix-Desmazes S Kaveri SV Bayry J. Surveillance of antigen-presenting cells by cd4+ Cd25+ Regulatory T cells in autoimmunity: immunopathogenesis and therapeutic implications. Am J Pathol. (2009) 174:1575–87. doi: 10.2353/ajpath.2009.080987 PMC2671245 19349365

[184] Pedroza-Pacheco I Madrigal A Saudemont A. Interaction between natural killer cells and regulatory T cells: perspectives for immunotherapy. Cell Mol Immunol. (2013) 10:222–9. doi: 10.1038/cmi.2013.2 PMC4012769 23524654

[185] Ohlsson M Hellmark T Bengtsson AA Theander E Turesson C Klint C. Proteomic data analysis for differential profiling of the autoimmune diseases sle, ra, ss, and anca-associated vasculitis. J Proteome Res. (2021) 20:1252–60. doi: 10.1021/acs.jproteome.0c00657 PMC7872503 33356304

[186] Alsaeed A. Comment on: autoimmune diseases and their prevalence in Saudi Arabian patients with type 1 diabetes mellitus. Saudi Med J. (2023) 44:1310. doi: 10.15537/smj.2023.44.12.20230818 38016741 PMC10712796

[187] Dmochowski M Jalowska M Bowszyc-Dmochowska M. Issues occupying our minds: nomenclature of autoimmune blistering diseases requires updating, pemphigus vulgaris propensity to affect areas adjacent to natural body orifices unifies seemingly diverse clinical features of this disease. Front Immunol. (2022) 13:1103375. doi: 10.3389/fimmu.2022.1103375 36601117 PMC9806572

[188] Boutzios G Koukoulioti E Goules AV Kalliakmanis I Giovannopoulos I Vlachoyiannopoulos P. Hashimoto thyroiditis, anti-parietal cell antibodies: associations with autoimmune diseases and Malignancies. Front Endocrinol (Lausanne). (2022) 13:860880. doi: 10.3389/fendo.2022.860880 35528009 PMC9072778

[189] McLarnon A. Ibd: regulatory T-cell therapy is a safe and well-tolerated potential approach for treating refractory Crohn’s disease. Nat Rev Gastroenterol Hepatol. (2012) 9:559. doi: 10.1038/nrgastro.2012.167 22926154

[190] Clough JN Omer OS Tasker S Lord GM Irving PM. Regulatory T-cell therapy in Crohn’s disease: challenges and advances. Gut. (2020) 69:942–52. doi: 10.1136/gutjnl-2019-319850 PMC7229901 31980447

[191] Diestelhorst J Junge N Schlue J Falk CS Manns MP Baumann U. Pediatric autoimmune hepatitis shows a disproportionate decline of regulatory T cells in the liver and of il-2 in the blood of patients undergoing therapy. PloS One. (2017) 12:e0181107. doi: 10.1371/journal.pone.0181107 28700730 PMC5507441

[192] Shin JI. The beneficial effect of leflunomide on systemic lupus erythematosus: the role of tregs repopulation?: comment on: leflunomide: friend or foe for systemic lupus erythematosus? Rheumatol Int. (2013) 33:273–6. doi: 10.1007/s00296-013-2795-z 23765200

[193] Shariati M Shaygannejad V Abbasirad F Hosseininasab F Kazemi M Mirmosayyeb O. Silymarin restores regulatory T cells (Tregs) function in multiple sclerosis (Ms) patients in vitro. Inflammation. (2019) 42:1203–14. doi: 10.1007/s10753-019-00980-9 30806958

[194] Zhang Y Wang HB Chi LJ Wang WZ. The role of foxp3+Cd4+Cd25hi tregs in the pathogenesis of myasthenia gravis. Immunol Lett. (2009) 122:52–7. doi: 10.1016/j.imlet.2008.11.015 19111574

[195] Chen Z Wang Y Ding X Zhang M He M Zhao Y. The proportion of peripheral blood tregs among the cd4+ T cells of autoimmune thyroid disease patients: A meta-analysis. Endocr J. (2020) 67:317–26. doi: 10.1507/endocrj.EJ19-0307 31827051

[196] Biton J Semerano L Delavallee L Lemeiter D Laborie M Grouard-Vogel G. Interplay between tnf and regulatory T cells in a tnf-driven murine model of arthritis. J Immunol. (2011) 186:3899–910. doi: 10.4049/jimmunol.1003372 21346237

[197] Chavele KM Ehrenstein MR. Regulatory T-cells in systemic lupus erythematosus and rheumatoid arthritis. FEBS Lett. (2011) 585:3603–10. doi: 10.1016/j.febslet.2011.07.043 21827750

[198] Grover P Goel PN Greene MI. Regulatory T cells: regulation of identity and function. Front Immunol. (2021) 12:750542. doi: 10.3389/fimmu.2021.750542 34675933 PMC8524049

[199] Negi S Saini S Tandel N Sahu K Mishra RPN Tyagi RK. Translating treg therapy for inflammatory bowel disease in humanized mice. Cells. (2021) 10:1847. doi: 10.3390/cells10081847 34440615 PMC8393385

[200] Jin J Liu X. Commentary: T cell metabolism: A new perspective on th17/treg cell imbalance in systemic lupus erythematosus. Front Immunol. (2023) 14:1164761. doi: 10.3389/fimmu.2023.1164761 37180112 PMC10167841

[201] Shao S Yu X Shen L. Autoimmune thyroid diseases and th17/treg lymphocytes. Life Sci. (2018) 192:160–5. doi: 10.1016/j.lfs.2017.11.026 29158050

[202] Dall’Era M Pauli ML Remedios K Taravati K Sandova PM Putnam AL. Adoptive treg cell therapy in a patient with systemic lupus erythematosus. Arthritis Rheumatol. (2019) 71:431–40. doi: 10.1002/art.40737 PMC6447289 30277008

[203] Sun J Yang Y Huo X Zhu B Li Z Jiang X. Efficient therapeutic function and mechanisms of human polyclonal cd8(+)Cd103(+)Foxp3(+) regulatory T cells on collagen-induced arthritis in mice. J Immunol Res. (2019) 2019:8575407. doi: 10.1155/2019/8575407 30915372 PMC6399536

[204] Barsheshet Y Wildbaum G Levy E Vitenshtein A Akinseye C Griggs J. Ccr8(+)Foxp3(+) T(Reg) cells as master drivers of immune regulation. Proc Natl Acad Sci U.S.A. (2017) 114:6086–91. doi: 10.1073/pnas.1621280114 PMC5468670 28533380

[205] Aricha R Reuveni D Fuchs S Souroujon MC. Suppression of experimental autoimmune myasthenia gravis by autologous T regulatory cells. J Autoimmun. (2016) 67:57–64. doi: 10.1016/j.jaut.2015.09.005 26489998

[206] Haque M Lei F Xiong X Das JK Ren X Fang D. Stem cell-derived tissue-associated regulatory T cells suppress the activity of pathogenic cells in autoimmune diabetes. JCI Insight. (2019) 4:e126471. doi: 10.1172/jci.insight.126471 30777937 PMC6483657

[207] Liu D Tu X Huang C Yuan Y Wang Y Liu X. Adoptive transfers of cd4(+) cd25(+) tregs partially alleviate mouse premature ovarian insufficiency. Mol Reprod Dev. (2020) 87:887–98. doi: 10.1002/mrd.23404 32741069

[208] Huang H Deng Z. Adoptive transfer of regulatory T cells stimulated by allogeneic hepatic stellate cells mitigates liver injury in mice with concanavalin a-induced autoimmune hepatitis. Biochem Biophys Res Commun. (2019) 512:14–21. doi: 10.1016/j.bbrc.2019.02.147 30853178

[209] Wang K Zhu T Wang H Yang J Du S Dong G. Adoptive transfers of cd4(+)Cd25(+) tregs raise foxp3 expression and alleviate mouse enteritis. BioMed Res Int. (2018) 2018:9064073. doi: 10.1155/2018/9064073 30364052 PMC6186320

[210] Jeon YW Lim JY Im KI Kim N Nam YS Song YJ. Enhancement of graft-versus-host disease control efficacy by adoptive transfer of type 1 regulatory T cells in bone marrow transplant model. Stem Cells Dev. (2019) 28:129–40. doi: 10.1089/scd.2018.0113 30381994

[211] Roemhild A Otto NM Moll G Abou-El-Enein M Kaiser D Bold G. Regulatory T cells for minimising immune suppression in kidney transplantation: phase I/iia clinical trial. BMJ. (2020) 371:m3734. doi: 10.1136/bmj.m3734 33087345 PMC7576328

[212] Abbaszadeh S Nosrati-Siahmazgi V Musaie K Rezaei S Qahremani M Xiao B. Emerging strategies to bypass transplant rejection via biomaterial-assisted immunoengineering: insights from islets and beyond. Adv Drug Delivery Rev. (2023) 200:115050. doi: 10.1016/j.addr.2023.115050 37549847

[213] Whitehill GD Amarnath S Muranski P Keyvanfar K Battiwalla M Barrett AJ. Adenosine selectively depletes alloreactive T cells to prevent gvhd while conserving immunity to viruses and leukemia. Mol Ther. (2016) 24:1655–64. doi: 10.1038/mt.2016.147 PMC5113112 27401140

[214] Handelsman S Overbey J Chen K Lee J Haj D Li Y. Pd-L1’s role in preventing alloreactive T cell responses following hematopoietic and organ transplant. Cells. (2023) 12:1609. doi: 10.3390/cells12121609 37371079 PMC10296912

[215] Cassady K Martin PJ Zeng D. Regulation of gvhd and gvl activity via pd-L1 interaction with pd-1 and cd80. Front Immunol. (2018) 9:3061. doi: 10.3389/fimmu.2018.03061 30622541 PMC6308317

[216] Huppert LA Green MD Kim L Chow C Leyfman Y Daud AI. Tissue-specific tregs in cancer metastasis: opportunities for precision immunotherapy. Cell Mol Immunol. (2022) 19:33–45. doi: 10.1038/s41423-021-00742-4 34417572 PMC8752797

[217] Beres AJ Drobyski WR. The role of regulatory T cells in the biology of graft versus host disease. Front Immunol. (2013) 4:163. doi: 10.3389/fimmu.2013.00163 23805140 PMC3690651

[218] Arjomandnejad M Kopec AL Keeler AM. Car-T regulatory (Car-treg) cells: engineering and applications. Biomedicines. (2022) 10:287. doi: 10.3390/biomedicines10020287 35203496 PMC8869296

[219] Muller YD Ferreira LMR Ronin E Ho P Nguyen V Faleo G. Precision engineering of an anti-hla-A2 chimeric antigen receptor in regulatory T cells for transplant immune tolerance. Front Immunol. (2021) 12:686439. doi: 10.3389/fimmu.2021.686439 34616392 PMC8488356

[220] MacDonald KG Hoeppli RE Huang Q Gillies J Luciani DS Orban PC. Alloantigen-specific regulatory T cells generated with a chimeric antigen receptor. J Clin Invest. (2016) 126:1413–24. doi: 10.1172/JCI82771 PMC4811124 26999600

[221] Dabrowska A Grubba M Balihodzic A Szot O Sobocki BK Perdyan A. The role of regulatory T cells in cancer treatment resistance. Int J Mol Sci. (2023) 24:14114. doi: 10.3390/ijms241814114 37762416 PMC10531820

[222] BaSudan AM. The role of immune checkpoint inhibitors in cancer therapy. Clin Pract. (2022) 13:22–40. doi: 10.3390/clinpract13010003 36648843 PMC9844484

[223] Marabelle A Kohrt HE Brody J Torchia JA Zhou G Luong R. Local treg immunomodulation cures metastatic lymphoma including cns sites. Blood. (2011) 118(21):LBA–2. doi: 10.1182/blood.v118.21.lba-2.bld0076_p1_lba-2

[224] Di Pilato M Kim EY Cadilha BL Prussmann JN Nasrallah MN Seruggia D. Targeting the cbm complex causes T(Reg) cells to prime tumours for immune checkpoint therapy. Nature. (2019) 570:112–6. doi: 10.1038/s41586-019-1215-2 PMC6656391 31092922

[225] Liu C Chikina M Deshpande R Menk AV Wang T Tabib T. Treg cells promote the srebp1-dependent metabolic fitness of tumor-promoting macrophages via repression of cd8(+) T cell-derived interferon-gamma. Immunity. (2019) 51:381–97 e6. doi: 10.1016/j.immuni.2019.06.017 31350177 PMC6703933

[226] Chow MT Luster AD. Chemokines in cancer. Cancer Immunol Res. (2014) 2:1125–31. doi: 10.1158/2326-6066.CIR-14-0160 PMC4258879 25480554

[227] Li C Jiang P Wei S Xu X Wang J. Regulatory T cells in tumor microenvironment: new mechanisms, potential therapeutic strategies and future prospects. Mol Cancer. (2020) 19:116. doi: 10.1186/s12943-020-01234-1 32680511 PMC7367382

[228] Maeda S Murakami K Inoue A Yonezawa T Matsuki N. Ccr4 blockade depletes regulatory T cells and prolongs survival in a canine model of bladder cancer. Cancer Immunol Res. (2019) 7:1175–87. doi: 10.1158/2326-6066.CIR-18-0751 31160277

[229] Wang H Franco F Tsui YC Xie X Trefny MP Zappasodi R. Cd36-mediated metabolic adaptation supports regulatory T cell survival and function in tumors. Nat Immunol. (2020) 21:298–308. doi: 10.1038/s41590-019-0589-5 32066953 PMC7043937

[230] Ward-Kavanagh LK Lin WW Sedy JR Ware CF. The tnf receptor superfamily in co-stimulating and co-inhibitory responses. Immunity. (2016) 44:1005–19. doi: 10.1016/j.immuni.2016.04.019 PMC4882112 27192566

[231] Muller D. Targeting co-stimulatory receptors of the tnf superfamily for cancer immunotherapy. BioDrugs. (2023) 37:21–33. doi: 10.1007/s40259-022-00573-3 36571696 PMC9836981

[232] Kremer J Henschel P Simon D Riet T Falk C Hardtke-Wolenski M. Membrane-bound il-2 improves the expansion, survival, and phenotype of car tregs and confers resistance to calcineurin inhibitors. Front Immunol. (2022) 13:1005582. doi: 10.3389/fimmu.2022.1005582 36618378 PMC9816406

